# Dual‐Function Bis‐Tetraphenylethenes for Selective Metal Ion and Glutathione Detection and Current Transformer Application

**DOI:** 10.1002/open.202500045

**Published:** 2025-05-06

**Authors:** Sinan Bayindir, Sebiha Akar, Abdullah S. Hussein, Ferruh Lafzi, İkram Orak

**Affiliations:** ^1^ Department of Chemistry Faculty of Sciences and Arts Bingöl University 12000 Bingöl Türkiye; ^2^ College of Education Chemistry Department Salahaddin University‐Erbil 44001 Erbil Iraq; ^3^ Department of Chemistry Faculty of Sciences Ataturk University 25240 Erzurum Türkiye; ^4^ Vocational School of Health Services Bingöl University 12000 Bingöl Türkiye

**Keywords:** optoelectronic, photovoltaic, rhodanine, sensors, tetraphenylethenes

## Abstract

This study reports the synthesis of bis‐substituted tetraphenylethenes (TPEs) and the investigation of their photophysical and photochemical properties. Utilizing the aggregation‐induced emission characteristic of TPEs, this study demonstrates that BPh‐TPE and BRh‐TPE function as “turn‐off” fluorescent sensors for detecting Cu^2+^ and Hg^2+^/Ag^+^, respectively, exhibiting quenched fluorescence in the presence of these ions. The limits of detection (LODs) for Cu^2+^, Hg^2+^, and Ag^+^ are determined to be 299, 522 nM, and 1.58 μM, respectively. Interestingly, the probes also show “turn‐on” fluorescence upon the addition of glutathione (GSH) in the presence of Cu^2+^ or Hg^2+^. Specifically, the LOD for GSH using the BPh‐TPE‐Cu^2+^ complex is 457 nM. Practical applicability is confirmed via visual color changes on filter paper and in water samples. These findings highlight the potential of these TPE derivatives for detecting copper, mercury, silver ions, and GSH in environmental water. Additionally, Al/organic layer/*p*‐Si heterojunction devices incorporating spin‐coated TPE layers are fabricated. Their electrical behaviors are analyzed through current‐voltage and current‐time measurements under varying light conditions. Transient photocurrent analysis indicates strong photoresponse, supporting their suitability for optoelectronic and photovoltaic applications.

## Graphical abstract

In this study, it develops mono/bis‐substituted tetraphenylethenes that exhibit unique photophysical properties, including aggregation‐induced emission. These properties make them promising candidates for various applications, such as ion sensing and organic electronics. This study successfully demonstrates their utility as a turn‐off and turn‐on fluorescent sensor for heavy metal ions (Cu^2+^, Hg^2+^, and Ag^+^) and glutathione.

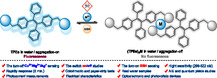

## 1 Introduction

Organic materials have also proven to be exciting platforms both in the area of sensing and optoelectronics, owing to their structural richness, low‐processing costs, and tunable electric and photonic properties.^[^ ^1^, ^2^, ^3^, ^4^ ^]^ Among these materials, the development of highly selective and sensitive fluorescent chemosensors toward metal and biologically important analytes has evoked significant interest. Recent advancements in the field of detection rely more on uncomplicated, one‐step, and easily observable techniques like fluorometric and colorimetric assays, which present easy, turnkey alternatives to instrument‐based detection.^[^ ^5^, ^6^, ^7^, ^8^, ^9^, ^10^, ^11^ ^]^ Heavy metals like mercury (Hg^2+^), copper (Cu^2+^), and silver (Ag^+^) are of special concern due to their environmental and cytotoxicity effects. Mercury is a highly toxic metal controlled by international health organizations, and the maximum tolerated levels are below 2 ppb in drinking water.^[^ ^12^, ^13^, ^14^ ^]^ Copper, though crucial in trace quantities in normal physiological processes, acts as a neurotoxin at toxic levels and is implicated in diseases like Alzheimer's and Parkinson's diseases.^[^ ^15^, ^16^, ^17^ ^]^ Growing industrial utilization of silver nanoparticles is problematic in terms of Ag^+^ ion release in the aquatic ecosystem.^[^ ^18^, ^19^, ^20^ ^]^ Likewise, glutathione (GSH), a tripeptoid and cellular antioxidant, plays a significant role in regulating redox homeostasis, and its abnormal levels are implicated in hepatotoxicity, cancer progression, and neuronal degeneration.^[^ ^21^, ^22^, ^23^, ^24^, ^25^, ^26^ ^]^ Tetraphenylethene (TPE)‐derived compounds have also attracted extensive interest owing to their distinct aggregation‐induced emission (AIE)‐based characteristics, which achieve fluorescence enhancement in aqueous or aggregate conditions.^[^ ^27^, ^28^, ^29^, ^30^ ^]^ These characteristics also render them prime candidates in creating “turn‐off” or “turn‐on” sensors that are readable in real‐world aqueous conditions. TPE derivatives also show high π‐conjugation and photostability, paving the way to use them as prospective building blocks in organic light‐emitting diodes and photovoltaics.^[^ ^31^, ^32^, ^33^ ^]^ While numerous reports exist on mono‐ or di‐substituted TPEs as selective metal ion sensors or as emitting units in optoelectronics,^[^ ^27^, ^28^, ^29^, ^30^, ^34^, ^35^, ^36^, ^37^ ^]^ there is limited research focused on the integration of these two aspects in one molecular system. For instance, Bayindir et al. presented the detection of metal ions using hydrazone‐linked TPEs,^[^ ^35^, ^37^ ^]^ and others investigated the TPE systems in the context of device manufacture. Nevertheless, the simultaneous testing of these materials as both reversible sensors and active interfacial layers in devices is quite limited. In the current work, we synthesized two bis‐substituted derivatives of TPE, BPh‐TPE and BRh‐TPE, with the functional units of the hydrazone and rhodanine type, respectively. These D–A systems were developed to provide multiple binding sites for the interaction of metal ions while keeping strong AIE ability. We examined the fluorescence response of the TPE systems toward a variety of cations and anions, paying special attention to the detection of Cu^2+^, Hg^2+^, Ag^+^, and GSH. Their high selectivity and low detection thresholds were confirmed in laboratory and real samples of water. Aside from solution‐phase detection, we integrated the TPEs in Al/TPE/*p*‐Si Schottky heterojunctions to study their effect on the transport of charges and photodetection under illumination conditions. To the best of our knowledge, the current work is the first attempt to merge the reversible and AIE‐based ion detection in a single TPE platform and solid‐state optoelectrical functionality in a single system. This work provides a starting point of the use of TPE materials in both environmental monitoring and organic electronic cells in a multifunctional manner.

## 2 Results and Discussion

### 2.1 Chemistry

**Scheme 1:**
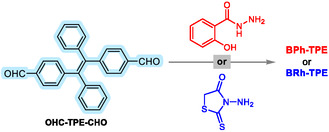
The synthesis strategy of **BPh‐TPE** and **BRh‐TPE.**

Recent research has focused on creating TPE‐based organic compounds and exploring their potential as ion sensors. This study contributes to this field by introducing two novel TPE derivatives with bis‐substitutions: **BPh‐TPE** and **BRh‐TPE**. We synthesized these compounds through a multistep reaction sequence (**Scheme** **1** and S1, Supporting Information). First, an intermediate, CHO‐TPE‐CHO, was prepared following a reported procedure (Scheme S1, Supporting Information).^[^ ^36^ ^]^ Subsequently, **BPh‐TPE** was obtained by reacting one equivalent of CHO‐TPE‐CHO with two equivalents of 2‐hydroxybenzohydrazide in refluxing ethanol. Similarly, **BRh‐TPE** was synthesized using 3‐amino‐2‐thioxothiazolidin‐4‐one (*N*‐amino‐rhodamine, Rh‐NH_2_) instead of Ph(OH)‐NH‐NH_2_. In addition, we investigated the photophysical properties of two monosubstituted TPEs (**Ph‐TPE** and **Np‐TPE**). These two molecules were resynthesized using a previously described method (Scheme S1, Supporting Information).^[^ ^27^ ^]^

### 2.2 Recognition Properties

**Figure 1:**
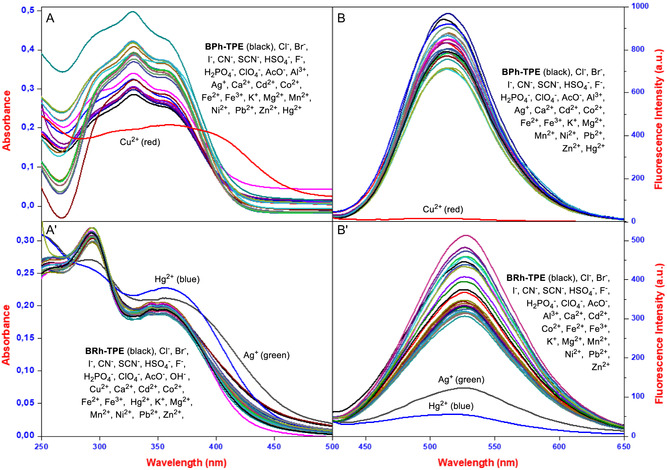
The UV‐Vis and fluorescence of spectrums A,B) BPh‐TPE and A′,B′) BRh‐TPE in the absence and presence of ions in water.

After synthesizing **BPh‐TPE** and **BRh‐TPE**, we explored their interaction with various ions through ultraviolet‐visible (UV‐Vis) and fluorescence spectroscopy in different solvent systems, including MeOH, EtOH, Dimethyl sulfoxide (DMSO), Tetrahidrofuran, their aqueous counterparts, and water. The probes’ interactions with a range of cations (Ag^+^, Al^3+^, Ca^2+^, Cd^2+^, Co^2+^, Cu^2+^, Fe^2+^, Fe^3+^, Hg^2+^, K^+^, Mg^2+^, Mn^2+^, Ni^2+^, Pb^2+^, and Zn^2+^ as their chloride salts) and anions ([Bu_4_N]F, [Bu_4_N]Cl, [Bu_4_N]Br, [Bu_4_N]I, [Bu_4_N]AcO, [Bu_4_N]HSO_4_, [Bu_4_N]ClO_4_, [Bu_4_N]CN, [Bu_4_N]SCN, [Bu_4_N]H_2_PO_4_, and [Bu_4_N]OH) were investigated across selected solvent systems. We initially tested the probes in various organic solvents (without water) to find the best environment for ion detection. No interaction between the probes and ions occurred in these pure solvents. However, both **BPh‐TPE** and **BRh‐TPE** probes specifically interacted with Cu^2+^ and Hg^2+^/Ag^+^ ions in water‐based solutions, including pure water and tap water. This indicates that both probes can effectively detect these metal ions in real water samples. Since water was chosen as the solvent system, we also investigated the ideal pH level for the probes’ interaction with metal ions. We tested a wide range (pH 2–12) and found that the probes interacted weakly with copper/mercury/silver ions in very acidic or basic environments. However, good interaction occurred at neutral pH 7 and in the 2‐[4‐(2‐Hydroxyethyl)piperazin‐1‐yl]ethane‐1‐sulfonic acid (HEPES) buffer. Surprisingly, changes in pH between 2 and 8 did not affect the probes. However, they did behave differently under more alkaline conditions (pH 9–12). Although working with real water samples offers advantages, various water ratios, and good reproducibility, the limited pH range for optimal interaction is a drawback. Following solvent and pH optimization, we examined how long the probes interacted with the target ions (copper, mercury, and silver) in the HEPES buffer (Figure S7A–C, Supporting Information). The probes only take about 5 min to reach maximum interaction, and this interaction remains stable over time (Figure S7D–F, Supporting Information). This is another significant advantage, as these probes show minimal interaction with other ions, even in long‐term studies, highlighting their selectivity. Finally, detailed data analysis revealed that pure water and real water samples, with or without HEPES buffer, are the most suitable environments for detecting copper, mercury, and silver ions using these probes. The UV‐Vis spectra for all ions were recorded for about 5 min following the addition of three equivalents of each ion. The spectral changes we observed confirmed that **BPh‐TPE** interacts with copper. That is, when Cu^2+^ was added, the peak absorbance of **BPh‐TPE** decreased from 327 to 335 nm with redshift (**Figure** **1**A). Similarly, the absorbance peak at ≈290 nm decreased, while that at 350 nm increased upon the addition of mercury or silver, shifting to 295 and 355 nm for Hg^2+^ and 295 and 360 nm for Ag^+^, respectively (Figure 1A′). Importantly, no significant changes were observed with other metal ions or common anions, except for the specific interactions mentioned above. In addition to UV‐Vis spectroscopy, we also investigated how **BPh‐TPE** and **BRh‐TPE** interact with various ions using fluorescence spectroscopy (Figure 1B/1B'). This technique confirmed the findings from the UV‐Vis studies, showing a specific response to Cu^2+^, Hg^2+^, and Ag^+^ ions. Following these initial tests, we conducted more detailed studies to assess how well these probes could detect ions in water (with or without HEPES buffer). When exposed to light with a wavelength of 400 nm, **BPh‐TPE** and **BRh‐TPE** emitted light at 510 and 520 nm, respectively. Adding most ions had little effect on this emitted light. However, a crucial finding was that Cu^2+^ ions interacted with **BPh‐TPE**, and Hg^2+^ or Ag^+^ ions interacted with **BRh‐TPE**, causing a significant decrease in the intensity of the emitted light.

This suggests that these probes have the potential to act as selective sensors for copper, mercury, and silver ions by turning off fluorescence in their presence. Understanding how individual ions interact with the probes is important, but real‐world water samples often contain multiple ions. Therefore, we conducted additional experiments to see if other ions would interfere with the detection of copper, mercury, and silver. These competitive experiments were done in water. The results were encouraging: The presence of other ions did not significantly affect the way the probes responded to Cu^2+^/Ag^+^ (Figure S8, Supporting Information). Moreover, the presence of iodine ions caused a different change in the spectrum of the BRh‐TPE+Hg^2+^ complex compared to the spectrum of BRh‐TPE. No significant change was observed with all other ions except iodine. This means that neither BPh‐TPE nor BRh‐TPE interaction with copper or silver was hindered by other ions present in pure water or real‐world water samples. On the other hand, treatment with glutathione (GSH), an important biological molecule, revealed that copper and mercury ions are bound to the probes, forming recoverable complexes. Interestingly, the BRh‐TPE‐Ag^+^ complex did not interact with GSH.

**Figure 2:**
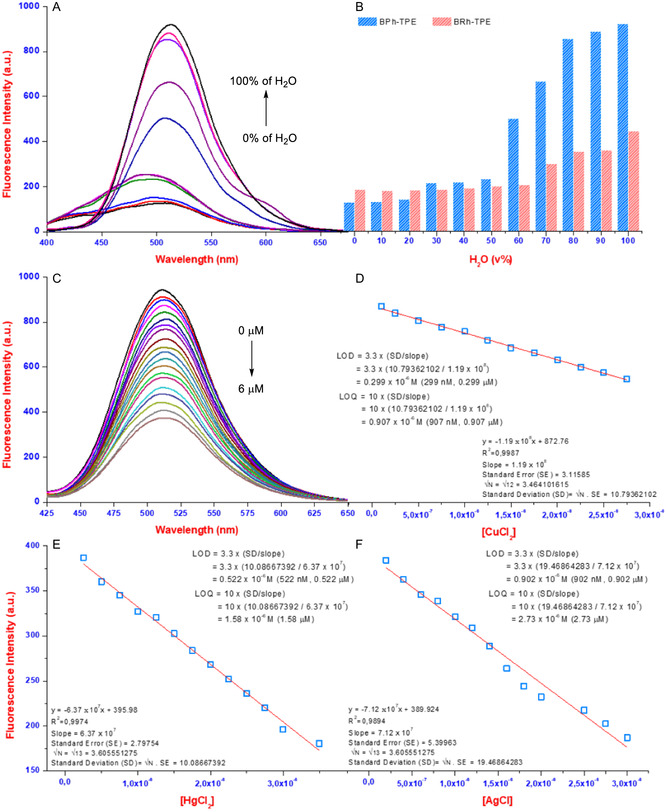
The A) AIE fluorescent response/B) bar graphic of BPh‐TPE/BRh‐TPE in different water ratio mixtures (λ_exc_ = 390 nm), C) the fluorescence titration of BPh‐TPE with CuCl_2_ and change fluorescence intensity of the D) BPh‐TPE/E,F) BRh‐TPE with the increasing concentration of ions.

Further investigation was needed due to a drop in fluorescence intensity observed after initial studies. This decrease could be linked to interactions between the ligands and ions. To explore this, we looked at AIE behavior (**Figure** **2**A,B and S9A, Supporting Information). The TPEs completely lost their fluorescence in pure ethanol. This quenching effect is likely due to photo‐induced electron transfer (PET) or excited‐state intramolecular proton transfer (ESIPT). Interestingly, adding water gradually (starting at 60% water) brought back fluorescence, reaching a peak at 100% water. This significant rise in intensity, along with a shift toward red wavelengths, confirms the AIE properties of the TPEs. The proposed mechanism involves the suppression of PET due to stronger aggregation in water. The presence of nitrogen and hydroxyl groups in the TPEs allows for electron transfer within the molecule, which is disrupted when aggregation occurs. Therefore, it is believed that hydrogen bonding between water molecules and the donor groups (hydroxyl, imine, or amine) on the TPEs hinders PET, leading to AIE. This AIE behavior was also observed in TPE‐M^+/2+^ complexes with increasing water content, but the increase in intensity was much smaller. This suggests that the altered structure of the complexes limits their ability to aggregate effectively. Complementing the AIE investigations, the fluorescence quantum yield (**Φ**) was calculated using the standard proportional method. To determine the quantum yield of the TPEs in an aqueous environment, rhodamine 101 (R101) in ethanol was employed as a reference standard with a normalized quantum yield of 1 at an excitation wavelength of 500 nm. The quantum yields of the sample (**Φ**_**r**_) and the ligand (**Φ**_**s**_) were determined using the following Equation (1)
(1)

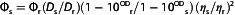




Here, ***D***_**r**_ and ***D***_**s**_ represent the corrected fluorescence spectrum areas of reference and ligand, respectively. The refractive indices of the solvents for the reference and ligand are denoted as **η**_**r**_ and **η**_**s**_, while **OD**_**r**_ and **OD**_**s**_ correspond to the optical densities of the reference and ligand at the excitation wavelength. Employing this method, the quantum yields of **BPh‐TPE** and **BRh‐TPE** were established to be 0.809 and 0.736, respectively, when excited at 370 nm (Figure S10, Supporting Information). Following, fluorescence titrations were performed for both **BPh‐TPE** and **BRh‐TPE** molecules to investigate their interaction with copper, mercury, and silver ions. As the metal concentration rose, the fluorescence intensity of both TPEs gradually decreased (quenched). This quenching reached a plateau at different levels for each metal ion: 6 μM for copper, 4 μM for mercury, and 4 μM for silver (Figure 2C and S9B,C, Supporting Information). The limit of detection (LOD) and limit of quantification (LOQ) for the TPEs were determined using the fluorescence titration data and specific equations (1 and 2, see supplementary data, Supporting Information). The calculated LOD and LOQ values for BPh‐TPE/Cu^2+^ are 299 and 907 nM, respectively. Similarly, the values for **BRh‐TPE** with Hg^2+^/Ag^+^ are 0.522/1.58 and 0.902/2.73 μM, respectively (Figure 2D–F). The ability of BPh‐TPE‐Cu^2+^ and BRh‐TPE‐Hg^2+^ complexes to reversibly bind with GSH ions suggested they could be useful as GSH sensors. To investigate this, fluorescence titration experiments were also conducted with increasing GSH concentrations. As expected, the fluorescence intensities at 510 and 520 nm increased proportionally with increasing GSH, reaching a plateau at around 4.0 μM (Figure S9D,E, Supporting Information). The LOD and LOQ values of GSH were calculated as 831 nM and 2.52 μM, respectively (Figure S9F, Supporting Information). These LOD and LOQ values indicate the high potential of these complexes for sensitive and accurate GSH detection. To further assess these probes as sensors, we determined their binding strength (affinity) for metal ions (Cu^2+^, Hg^2+^, and Ag^+^) using a technique called a Job's plot experiment (details in the Experimental Section). This experiment involved measuring the fluorescence intensity of mixtures containing different ratios of probes to metal ions. The results showed that the probes form a 2:1 complex (two probes binding to one metal ion) with the metal ions (Figure S11 and S12, Supporting Information). Next, we calculated the binding constant (K_a_) for each metal ion using the fluorescence titration data. The *K*_a_ values for Cu^2+^, Hg^2+^, and Ag^+^ were found to be 2.45 × 10^3^, 4.57 × 10^2^, and 5.80 × 10^2^ m ^‐1/2^, respectively (Figure S13, Supporting Information). These high *K*_a_ values indicate a relatively strong binding affinity between the probes and the metal ions, making them promising candidates for metal ion sensors.

**Figure 3:**
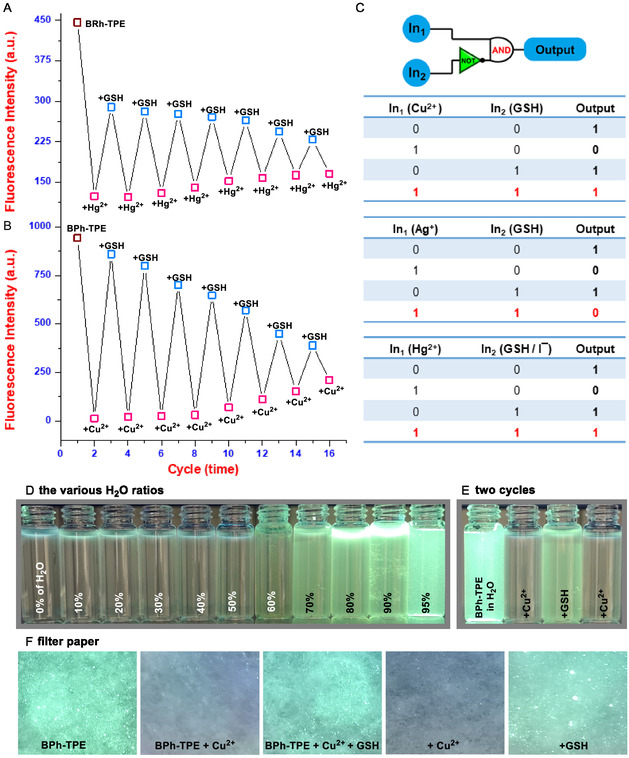
The reversible switching of the fluorescence intensity of A) BRh‐TPE/B,C) BPh‐TPE the “IMPLICATION” logic gate. D) The photographic images of BPh‐TPE in the various H_2_O ratios. E) the color changes of BPh‐TPE upon alternate addition of Cu^2+^/GSH for two cycles under UV light and F) the color changes on test paper of BPh‐TPE upon alternate addition of Cu^2+^ and GSH for two cycles under UV light.

One key advantage of these organic probes is their ability to turn fluorescence on and off in response to specific analytes. Experiments showed that adding Cu^2+^/Hg^2+^ ions and then GSH to the probes (**BPh‐TPE** and **BRh‐TPE**) caused the fluorescence at 510 and 520 nm to switch on and off repeatedly. Interestingly, this recycling only worked for Cu^2+^/Hg^2+^ and GSH, not for silver ions. This reusability allows the probes to be used for Cu^2+^/Hg^2+^ and GSH detection for up to 16 cycles (**Figure** **3**A,B and S14, Supporting Information). The interaction between the probes and metal ions appears to form a stable complex with GSH, as adding more GSH caused the fluorescence to decrease. This unique property of the probes allowed us to explore their potential for building a molecular logic gate. In this logic system, “on” and “off” fluorescence states were assigned the values “1” and “0,” respectively. When no metal ions (Input 1) or GSH (Input 2) were present, the probes remained “on” (output logic “1”). However, adding metal ions (In1) turned the fluorescence “off” (output logic “0”). Conversely, adding GSH (In2) to BPh‐TPE‐Cu^2+^ and BRh‐TPE‐Hg^2+^ switched the fluorescence “on” (output logic “1"). This response was not observed for BRh‐TPE‐Ag^+^ with GSH, which remained “off” (output logic “0”) (Figure 3C). Additionally, simple techniques like colorimetric tests and filter paper assays can effectively complement sophisticated instruments for evaluating sensor candidates. We used these methods along with real water samples to analyze our promising sensor candidates. Interestingly, the fluorescence intensity of the **BPh‐TPE** increased as the water content rose, a phenomenon known as AIE. This behavior likely stems from reduced electron transfer when the **BPh‐TPE** disperses (disaggregates) in water due to the presence of nitrogen (N), sulfur (S), and oxygen (O) groups within their structure (Figure 3D). Adding Cu^2+^ ions quenched the fluorescence, while adding glutathione (GSH) restored it, confirming reversible binding (Figure 3E). Finally, we explored practical applications by dipping filter paper strips in the **BPh‐TPE** solution. When dipped in solutions containing both Cu^2+^ ions and GSH, these strips displayed rapid and visible color changes under UV light (Figure 3F). This simple and affordable method offers a user‐friendly way to detect specific Cu^2+^ ions and GSH, highlighting the potential of **BPh‐TPE** for developing “on‐off‐on” sensors. The first part of this study explored bis‐substituted TPEs for detecting copper, mercury, silver, and glutathione in water. To demonstrate the usability of our findings, we compared our sensor candidates to existing ones for detecting copper, mercury, silver, and glutathione, focusing on binding strength and LOD. The data presented in **Table** **1** shows that our sensors achieve comparable performance, indicating their potential for water analysis.^[^ ^29^, ^33^, ^36^, ^37^, ^38^, ^39^, ^40^, ^41^, ^42^, ^43^, ^44^, ^45^, ^46^, ^47^, ^48^, ^49^ ^]^ Moreover, the TPE derivatives synthesized in this study offer valuable additional features: effective LOD values, recyclability, and conditional multi‐ion selectivity, making them stand out. The ability to adjust selectivity through side group changes in a consistent core structure is also theoretically important.

**Table 1:** Comparison of some Cu^2+^, Hg^2+^, Ag^+^, and GSH selective chemosensors.

Reference	Sensing ions	LOD
[12]	Cu^2+^	2.42 μM
	Hg^2+^	390 nM
[27]	Cu^2+^	597 nM
	GSH	544 nM
[30]	Cu^2+^	15.7 μM
	Hg^2+^	18.0 μM
[34]	Hg^2+^	0.41 μM
[44]	Cu^2+^	1.60 μM
[45]	Cu^2+^	10.0 μM
[46]	Cu^2+^	7.60 μM
[47]	Hg^2+^	0.10 μM
[48]	Cu^2+^	15.7 μM
	Hg^2+^	18.0 μM
[49]	Ag^+^	0.13 μM
[50]	Ag^+^	3.15 μM
[51]	Ag^+^	2.26 μM
[52]	Ag^+^	3.91 μM
[53]	GSH	4.30 μM
[54]	GSH	30.0 nM
[55]	GSH	37.0 nM
This study	Cu^2+^	289 nM
	Hg^2+^	522 nM
	Ag^+^	1.58 μM
	GSH	457 nM

### 2.3 Characterization and Electrical Properties

Interaction morphology significantly influences sensor and transistor performance. This study initially investigated BPh‐TPE and BRh‐TPE as potential selective ion binders. Literature suggests three possible binding sites within TPEs: the Schiff base nitrogen, aryl hydroxyl groups, and linker amine core. Due to the paramagnetic nature of the copper complex, ^1^H‐NMR spectroscopy was ineffective in studying the interaction. On the other hand, the interaction of BRh‐TPE with mercury ions was elucidated by ^1^H‐NMR titration. Accordingly, significant spectral changes, including peak shifts and the formation of new peaks, indicated the formation of a stable complex. These alterations were attributed to the interaction of mercury ions with the N and S groups within the rhodanine ring of BRh‐TPE. Specifically, the CH_2_ protons in the rhodanine ring and the enamine (HC=N—) peaks exhibited notable shifts and new peak formation, consistent with a change in the electronic environment due to complexation (Figure S15, Supporting Information). Following spectroscopic studies, the bandgap energies (*E*_g_) of the TPEs‐based devices (D1–D3) were also experimentally determined through UV‐Vis spectroscopy. To calculate *E*_g_, the absorption coefficient (α) was first derived from the film thickness (*d*) and optical transmittance (*T*) using Equation (2).

(2) α=ln(1−T)/d

Subsequently, *E*_g_ was calculated using Equation (3), where *hν* represents photon energy and *K* is a material constant.^[^ ^19^ ^]^

(3) $$\alpha h\nu =K{\left(h\nu -E_{g}\right)}^{2}$$

**Figure 4:**
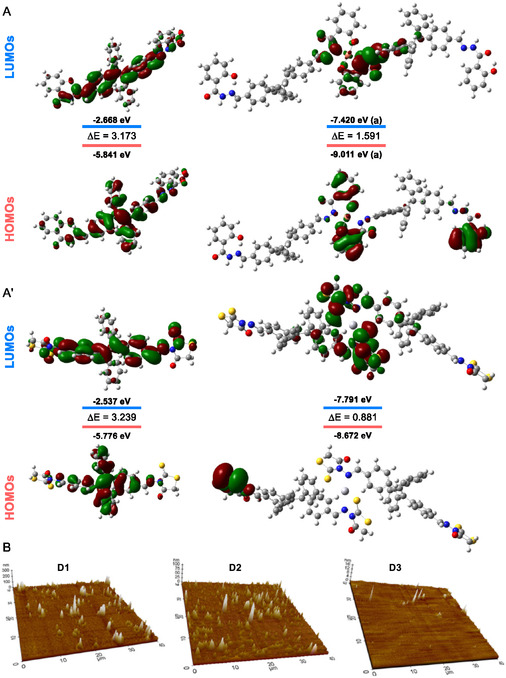
The HOMO/LUMO orbital distributions of A) BPh‐TPE/A',B) BRh‐TPE 3D‐AFM morphology of TPE‐based devices (D1–D3).

The obtained *E*_g_ values for D1, D2, and D3 were −3.08, −2.97, and −2.98 eV, respectively (Figure S16, Supporting Information). Notably, a wider HOMO‐LUMO gap, as reflected in a higher Eg, correlates with increased chemical stability, while a narrower gap implies greater reactivity. Given their experimental *E*_g_ values and absorbance profiles, these devices show potential for photovoltaic applications. Moreover, the energy gap (*E*_g_) values of TPEs were calculated theoretically. For this purpose, initially, the optimized geometries, the highest occupied molecular orbital (HOMO), and lowest unoccupied molecular orbital (LUMO) energies of TPEs, along with their complexes with Hg^2+^ and Cu^2+^, were determined using the DFT‐B3LYP method at the Density Functional Theory (DFT).^[^ ^50^ ^]^ The LanL2DZ basis set was applied to Hg^2+^ and Cu^2+^ ions, while the 6‐311G(d,p) basis set was used for the main group elements (**Figure** **4**A, S17, and S18, Supporting Information). The time‐dependent DFT (TD‐DFT) calculations at the same level produced the corresponding frontier molecular orbital (MOs) results. The calculated *E*_g_ values in the gas phase were consistent with the experimental values. Despite being small organic molecules, these results suggest that TPEs possess suitable electronic properties for potential applications in electrical and photophysical devices. The low *E*_g_ values and the observed absorbance in the UV‐Vis spectra indicate that TPEs could be promising candidates for these applications. To gain deeper insights into the electronic structure of TPEs, we calculated various quantum chemical descriptors, including *E*_g_, ionization potential (*I*), electron affinity (*A*), hardness (*η*), softness (*ς*), electronegativity (*χ*), chemical potential (*μ*), electrophilicity index (*ω*), nucleophilicity index (*N*), maximum charge transfer index (Δ*N*max), and optical softness (*σo*) (**Table** **2**).^[^ ^11^ ^]^ Domingo et al. proposed an electrophilic index (*ω*) scale to classify organic compounds based on their electrophilicity.^[^ ^51^ ^]^ According to this scale, compounds with *ω* > 1.5 eV are strong electrophiles, those with 0.8 < *ω* < 1.5 eV are moderate electrophiles, and those with *ω* < 0.8 eV are weak. In this particular, the TPEs in our study exhibit acceptable binding capacities, with electrophilic index values ranging from 13.7902 to 15.9012 eV (Table 2, Entry 13). Furthermore, we fabricated Schottky devices D1–D3 based on TPEs to investigate their photophysical properties and characterize their performance. After NDIs were coated on *p*‐Si, the substrate surface morphologies were scanned 40 × 40 μm with atomic force microscopy, as can be seen in both 2D and 3D images in Figure 4B. When the AFM results were analyzed, the root means square (RMS) roughness values of D1, D2, and D3. The RMS values were 9.3 and 11.4 nm for D1, 2.4, and 5.4 nm for D2, and 0.23 and 0.35 nm for D3. Surface roughness affects the device's performance under light (Figure S19, Supporting Information). The choice of solvent for the organic material and experimental optimizations significantly minimize roughness values for these devices. The thickness of the thin film affects some electrical properties, such as photocurrent, series resistance, and charge storage.

**Table 2:** Some chemical parameters of TPEs computed by DFT methods.

	BPh‐TPE	BPh‐TPE‐Cu [α]	BPh‐TPE‐Cu [β]	BRh‐TPE	BRh‐TPE‐Hg	Ph‐TPE	Np‐TPE
E_HOMO_	–5.841	–9.011	–8.659	–5.776	–8.672	–5.716	–5.991
E_LUMO_	–2.668	–7.42	–8.373	–2.537	–7.791	–2.15	–1.806
ΔE	3.173	1.591	0.286	3.239	0.881	3.566	4.185
μ	–4.2545	–8.2155	–8.516	–4.1565	–8.2315	–3.933	–3.8985
ζ	0.6303	1.2571	6.9930	0.6175	2.2701	0.5609	0.4779
σ_o_	0.3152	0.6285	3.4965	0.3087	1.1351	0.2804	0.2389
χ	4.2545	8.2155	8.516	4.1565	8.2315	3.933	3.8985
EA	2.668	7.420	8.373	2.537	7.791	2.15	1.806
IP	5.841	9.011	8.659	5.776	8.672	5.716	5.991
η	1.5865	0.7955	0.143	1.6195	0.4405	1.783	2.0925
N	0.1753	0.0236	0.0039	0.1875	0.0130	0.2305	0.2754
ΔN_max_	2.6817	10.3275	59.5524	2.5665	18.6867	2.2058	1.8631
ω	14.3584	26.8459	5.1853	13.9896	14.9236	13.7902	15.9012

### 2.4 Photovoltaic Studies

**Figure 5:**
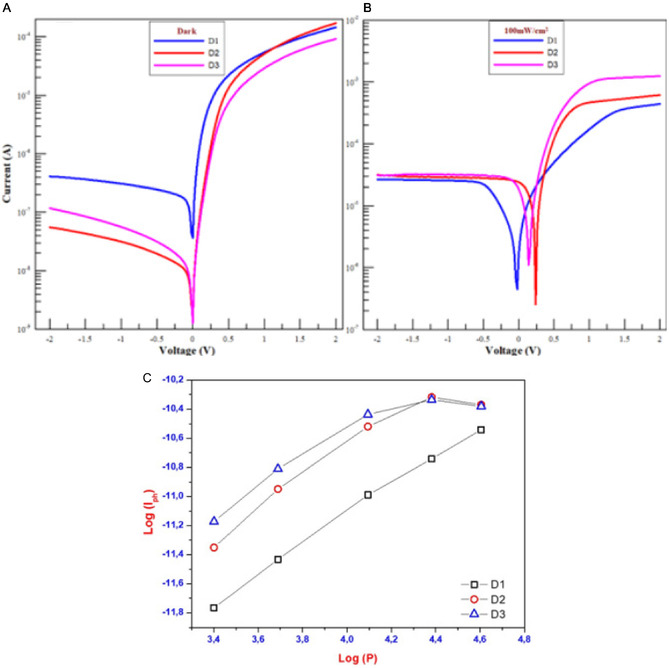
The comparison of the current value of devices A) at dark, B) under 100 mW cm^−2^, and C) the log(*I*_ph_) − log(P) plot of all SDs in various illumination intensities at −2 V.

After synthesizing TPEs, examining photochemical properties, and analyzing the surface morphology of devices incorporating these derivatives, photophysical studies of the devices were performed. In this context, **Figure** **5** shows graphs of devices with interfacial layers, which are D1, D2, and D3 at dark and under 100 mW cm^−2^. As can be seen in Figure 5A, especially reverse bias region (*V* < 0), there is an ≈100 times increase in the current values of devices with interface layers, but not at the forward bias region (*V* > 0) at dark conditions. In these measurements, it is observed that D2 and D3 have similar performance, while D1 has poorer IV values compared with others. In Figure 5B, all devices have a response to the light under illumination conditions. The change in the IV values of the devices under illumination conditions is that they are photovoltaic devices. Additionally, all devices have an almost good rectifying behavior (RB). In Table 3, the RB values of D1, D2, and D3 are about 344, 3036 and 775, respectively, in dark conditions. The characteristics of Schottky diode and photovoltaic devices are examined using the thermionic emission theory.^[^ ^52^ ^]^ In this theory, the main electrical parameters, such as the ideality factor (*n*), the saturation current (*I*_0_), and the barrier height (*Φ*_B0_) of the device are decided by using Equation (4) and (5) as well as data in the region corresponding to the linearity behavior of the *I–V* plot of the devices.

(4) $$I=\left\{AA^{*}T^{2}\exp\left(-\frac{q}{kT}\Phi_{B0}\right)\right\}\left[\exp\left(\frac{q\left(V-IR_{s}\right)}{nkT}\right)-1\right]$$

(5) $$\Phi_{B0}=\frac{kT}{q}\text{ln}\left(\frac{AA^{*}T^{2}}{I_{0}}\right)$$

(6) $$n=\frac{q}{kT}\left(\frac{dV}{d(\ln(I))}\right)$$

where A*, *A*, *T*, *V*, and *R*_s_ denote Richardson's constant, the area of the device, the absolute temperature in Kelvin, the applied bias voltage, and series resistance of the device, respectively. The *n*, *I*_0_, and *Φ*_B0_ values calculated for the devices at dark and under illumination intensities are given in **Table** **3**. As shown in this table, the n values for all SDs are higher than the ideality value (=1). This can be due to the surface state, interface layer, depletion region width, and device manufacturing defects.^[^ ^53^ ^]^ Among these devices, the D1 device has the best ideality factor. As can be seen in Table 3, the higher *Φ*_B0_ value among devices with an organic interfacial layer in the dark is D1. In general, the devices have resistance, which are the series (RS), and the shunt (RSH) (Figure S20, Supporting Information). These parameters affect the current characteristics of the diode and photodiode. The Rs is resistance at forward bias, and the Rsh is at reverse bias. The values of both Rs and Rsh can be determined by using Ohm's Law (=d*V*/d*I*).^[^ ^52^ ^]^ The values of Rs and Rsh calculated by using Ohm's Law at dark and under illumination intensities at +2 and −2 V, respectively, are given in Table 3. The Rs has a high value due to Ohmic contact, the interface layer's presence, and the depletion layer's width.^[^ ^54^ ^]^

**Table 3:** The *n*, *I*_0_, *Φ*_B0_ *R*_s_, *R*_sh_, and RB values calculated for the SDs at dark and under illumination intensities.

Devices	Power [mW cm^−2^]	*n*	*I*_0_ [A]	*Φ*_B0_ [eV]	*R*_s_ (+2 V) [W]	*R*_sh_ (−2 V) [W]	RB
D1	Dark	1.83	1.61 × 10^7^	0.68	13944	4.8 × 10^6^	344.2
	100	10.3	1.23 × 10^5^	0.55	4500	75653	16.8
D2	Dark	2.2	9.98 × 10^9^	0.73	11857	3.6 × 10^7^	3036.1
	100	2.5	1.14 × 10^7^	0.67	3230	63758	19.7
D3	Dark	2.4	1.71 × 10^8^	0.72	21930	1.7 × 10^7^	775.1
	100	4.07	2.26 × 10^6^	0.55	1596	64434	40.3

The following equation can express the relationship between photocurrent (Iph) and the applied illumination intensity, which corresponds to the photosensitivity Equation (7).^[^ ^55^, ^56^ ^]^

(7) $$I_{\text{ph}}=\alpha P^{m}$$

where I_ph_, *α*, *P*, and m denote photo‐generated current, α constant, the illumination intensity, and an exponent, respectively. The m equals to slope of the log(*I*_ph_) ‐ log(P) plot (Figure 5C) and determines whether PM has a linear (*m* < 1) or nonlinear behavior (*m* > 1). The m values for the SDs with D1, D2, and D3 interfacial layers were calculated as 1.08, 0.85, and 0.62 at −2 V, respectively.^[^ ^57^ ^]^ These m results show that all SDs have a linear behavior. That indicates the existence of the localized states within the bandgap.^[^ ^55^, ^58^ ^]^ In photovoltaic devices, the mmm value (the exponent expressing the relationship between light intensity and photovoltaic current) is generally close to 1, but in some cases, it can be greater than 1. This depends on the device's operating conditions, material properties, and the carrier mechanisms induced by light. When the mmm value is greater than 1, it usually indicates the presence of nonlinear carrier processes or multiphoton absorption in the photovoltaic cell. For example, the recombination or trapping processes of carriers generated by photons can lead to mmm being greater than 1. Additionally, exciton dissociation, trap states, or the heterogeneous structure of the photovoltaic device can also influence this value.

There are some parameters determined for photosensitive and photodetect characteristics such as spectral responsivity (*R*), detectivity (D*), noise equivalent power (NEP), and spectral external quantum efficiency (EQE). These parameters are defined by Equation (8)–(11), respectively.^[^ ^59^, ^60^ ^]^

(8) $$R=\frac{I_{\text{lph}}-I_{\text{dark}}}{P_{\text{in}}A}$$

(9) $$D^{*}=R\sqrt{\frac{A}{2qI_{\text{dark}}}}$$

(10) $$\text{NEP}=\frac{\sqrt{A}}{D^{*}}$$

(11) $$\text{EQE}=R\frac{hc}{q\lambda }$$

where *A*, *h*, *c*, and l denote the surface area of the SD, the Planck constant, the speed of light in vacuum, and wavelength, respectively. The *R* is the ratio of the photocurrent produced to illumination intensities. The D* is the capability of detecting the incident light. NEP is the amount of input illumination power required to achieve a signal‐to‐noise ratio of one within a bandwidth of 1 Hz. The EQE is the number of electron–hole pairs generated per absorbed photon. The values of *R*, D*, NEP, and EQE were calculated for all SDs at −2 V under various illuminations in **Table** **4**. According to *I–V* measurements, the photovoltaic and photodetector performance of D2 is better than D1 and D3. As can be seen in Table 4, NEP and EQE values are very suitable for photodetector applications.

**Table 4:** Some detect parameters for all SDs at −2 V under various illumination intensities.

Interfacial layer	Power [mW cm^−2^]	*R* [A W^−1^]	D* [Jones]	NEP [W Hz^−1/2^]	EQE
D1	30	3.12 × 10^−2^	7.66 × 10^9^	1.16 × 10^−11^	10.64
	40	3.32 × 10^−2^	8.14 × 10^9^	1.09 × 10^−11^	11.31
	60	3.50 × 10^−2^	8.57 × 10^9^	1.03 × 10^−11^	11.91
	80	3.38 × 10^−2^	8.28 × 10^9^	1.07 × 10^−11^	11.50
	100	3.32 × 10^−2^	8.13 × 10^9^	1.09 × 10^−11^	11.30
D2	30	4.97 × 10^−2^	3.31 × 10^10^	2.68 × 10^−11^	16.92
	40	5.58 × 10^−2^	3.71 × 10^10^	2.39 × 10^−11^	19.00
	60	5.72 × 10^−2^	3.81 × 10^10^	2.33 × 10^−11^	19.48
	80	5.25 × 10^−2^	3.50 × 10^10^	2.53 × 10^−11^	17.90
	100	3.99 × 10^−2^	2.66 × 10^10^	3.34 × 10^−11^	13.59
D3	30	5.92 × 10^−2^	2.70 × 10^10^	3.28 × 10^−11^	20.16
	40	6.39 × 10^−2^	2.92 × 10^10^	3.03 × 10^−11^	21.77
	60	6.21 × 10^−2^	2.83 × 10^10^	3.12 × 10^−11^	21.15
	80	5.15 × 10^−2^	2.35 × 10^10^	3.77 × 10^−11^	17.53
	100	3.94 × 10^−2^	1.80 × 10^10^	4.92 × 10^−11^	13.42

## 3 Conclusion

In conclusion, this work effectively illustrates the dual utility of two rationally synthesized bis‐substituted TPEs derivatives, **BPh‐TPE** and **BRh‐TPE**. These compounds show different AIE characteristics and serve as highly selective and sensitive fluorescent probes for the detection of Cu^2+^, Hg^2+^, Ag^+^, and GSH in water systems. Most importantly, the “turn‐off/turn‐on” fluorescence switchable by reversible ion‐GSH interactions indicates high reusability potential. These TPEs proved useful as selective fluorescent probes for the detection of ions as turn‐off sensors of Cu^2+^ (299 nM), Hg^2+^ (522 nM), and Ag^+^ (1.58 μM). The probes also displayed a turn‐on fluorescence response toward GSH (457 nM, using BPh‐TPE‐Cu^2+^) in the presence of copper or mercury ions. In addition, these materials were also used in Al/TPE/*p*‐Si heterojunctions, where their interfacial properties led to high levels of photocurrent generation and positive device parameters under illumination. This interdisciplinary study emphasizes the versatility of TPE‐based materials in molecular sensing and solid‐state devices. While the probes exhibit good performance in neutral aqueous solutions, their selectivity in multi‐ion matrices and sensitivity in acidic and alkaline conditions need optimization. Device lifetime and long‐term operating stability were not in the scope of the present work. Future work will entail structural tuning to increase ion selectivity, integration in flexible electronic systems, and in vivo biosensing uses.

## 4 Experimental Section

4.1

#### Synthesis of TPEs

The target probes, **BPh‐TPE** and **BRh‐TPE**, were synthesized through a two‐step process (Scheme 1 and S2, Supporting Information). The starting material, CHO‐TPE‐CHO, was prepared using a previously reported method.^[^ ^35^ ^]^ In the first step, CHO‐TPE‐CHO (100 mg, 0.26 mmol) was dissolved in 15 mL of ethanol. Then, either 2‐hydroxybenzohydrazide (78.0 mg, 0.52 mmol) for **BPh‐TPE** or 3‐amino‐2‐thioxothiazolidin‐4‐one (76.3 mg, 0.52 mmol) for **BRh‐TPE** was added to the reaction mixture. The mixtures were heated under reflux overnight (at boiling temperature) and subsequently cooled to room temperature. Following filtration, the crude products were recrystallized from ethanol to yield pure **BPh‐TPE** and **BRh‐TPE** as solids. The reaction afforded **BPh‐TPE** and **BRh‐TPE** an 85% and 80% yield, respectively. Detailed experimental procedures and analysis data for the products (spectroscopic data) are available in the Supporting Information. **BPh‐TPE**: ^1^H NMR (400 MHz, DMSO‐d6) δ 11.86 (bs, OH, NH, 4H), 8.36 (s, N=CH, 2H), 7.88 (m, =CH, 2H), 7.43–7.52 (m, =CH, 8H), 6.96–7.16 (m, =CH, 16H); ^13^C NMR (100 MHz, CDCl_3_) δ 165.19, 159.61, 148.73, 145.54, 143.09, 141.32, 134.30, 132.88, 131.66, 131.24, 129.03, 128.54, 128.45, 127.22, 119.35, 117.78, 116.35 (Figure S3, Supporting Information). **BRh‐TPE**: ^1^H‐NMR (400 MHz, DMSO‐d6): δ 8.61 (s, N=CH, 2H), 7.75–7.63 (m, =CH, 8H), 7.21–6.96 (m, =CH, 10H), 4.34 (s, CH_2_, 4H); ^13^C‐NMR (100 MHz, DMSO‐d6): δ 197.5, 171.0, 170.3, 148.5, 142.8, 141.8, 132.2, 131.4, 13.8, 130.7, 129.2, 128.8, 127.8, 35.4 (Figure S4, Supporting Information). Additionally, the synthesis of monosubstituted‐TPEs **Ph‐TPE** and **Np‐TPE**, for which photophysical studies were carried out in this study, was carried out as described in the literature and is given in detail in Supporting Information.^[^ ^37^ ^]^

#### Procedures of Photophysical Measurement

To evaluate the ion‐sensing properties of **BPh‐TPE** and **BRh‐TPE**, UV‐Vis and fluorescence spectra were recorded in water at room temperature. The experiments involved adding various metal ions to the TPE solutions one at a time. Separate fluorescence titrations were conducted for **BPh‐TPE** with CuCl_2_ and **BRh‐TPE** with HgCl_2_/AgCl, respectively. These titrations involved adding increasing concentrations of the metal ion solutions to the TPE solutions in both pure water and real water samples. All measurements were repeated for consistency. Job's plot analysis was employed to determine the binding stoichiometry between the TPEs and the target ions. The LOD, LOQ, and *K*_a_ values were calculated using related formulas. Detailed experimental procedures can be found in Supporting Information.

#### Devices Fabrication

To fabricate the photodiode, the *p*‐Si with a thickness of 525 mm and resistivity of 1–10 Ω cm was subjected to a chemical cleaning process. Here, p‐Si was cleaned for 5 min at +30 °C in deionized water, acetone, isopropyl alcohol, and again deionized water, respectively. Then, p‐Si was dried with N_2_. In the following stage, the Al Ohmic contact was formed on the back side of p‐Si in the thermal evaporation system. To form an interfacial layer, the extracted NDIs were carried out on the front side of p‐Si at doping for 60 min. In the final stage, the Au rectifier contacts with a diameter of 1 mm and a thickness of 1500 Å were formed on the interfacial layer‐coated side of *p*‐Si in a thermal evaporation system (Figure S21, Supporting Information).

## Conflict of Interest

The authors declare no conflict of interest.

## Supporting information

Supplementary Material

## Data Availability

The data that support the findings of this study are available in the supplementary material of this article.
