# Supplementary material for: Dual‐Function Bis‐Tetraphenylethenes for Selective Metal Ion and Glutathione Detection and Current Transformer Application
Source: ChemistryOpen. 2025 May 6;14(9):e202500045. doi: 10.1002/open.202500045 (PMC12409823; doi:10.1002/open.202500045)
Supplement: Supplementary file 1 — Supplementary Material [file OPEN-14-e202500045-s001.pdf]

Supporting Information

Dual-Function Bis-Tetraphenylethenes for Selective Metal Ion and Glutathione Detection and Current Transformer Application

Sinan Bayindir[a*], Sebiha Akar[a], Abdullah S. Hussein[a,b], Ferruh Lafzi*[c] and İkram Orak[d]

[a]*Department of Chemistry, Faculty of Sciences and Arts, Bingol University, 12000, Bingol, Türkiye*
[b]*College of Education Chemistry Department, Salahaddin University-Erbil, 44001, Erbil, Iraq*
[c]*Department of Chemistry, Faculty of Sciences, Ataturk University, 25240, Erzurum, Türkiye*
[d]*Vocational School of Health Services, Bingol University, 12000, Bingol, Türkiye*

*Corresponding author:

E-mail address: *sbayindir@bingol.edu.tr, farrokh.lafzi@atauni.edu.tr*

General methods

All chemicals, reagents, and solvents were commercially available from Sigma-Aldrich or Merck. ^{1}H NMR and ^{13}C NMR spectra were recorded on a 400 (100)-MHz Bruker spectrometer and are reported in terms of chemical shift (δ, ppm) with $SiMe_4$ as an internal standard. Data for ^{1}H NMR are recorded as follows: chemical shift (δ, ppm), multiplicity (s: singlet, d: doublet, t: triplet, q: quartet, p: pentet, m: multiplet, bs: broad singlet, bd: broad doublet, qd: quasi doublet) and coupling constant (s) in Hz, integration. Elemental analyses were carried out on a LECO CHNS-932 instrument. Column chromatography was performed on silica gel 60 (230–400 mesh ASTM). The reaction progress was monitored by thin-layer chromatography (TLC) (0.25-mm-thick precoated silica plates: Merck Fertigplatten Kieselgel (60 F254)). UV-Vis absorption and fluorescence spectra of samples were recorded on a Shimadzu UV-3101PL UV-Vis-NIR spectrometer and Perkin–Elmer (Model LS 55) Fluorescence Spectrophotometer, respectively.

Synthesis of TPEs

*1,2-Bis(4-bromophenyl)-1,2-diphenylethene (**2**)***:** Compound **2** was synthesized according to the literature methods.[1-3] The 4-bromobenzophenone (**1**, 1.0 g, 3.30 mmol) and zinc dust (625 mg, 9.6 mmol) into a 250 mL two-necked round-bottom flask condenser were stirred at reflux temperature. The reaction mixture was evacuated under vacuum and pumped in dry nitrogen three times, and then 30 mL of dry THF was added. The $TiCl_4$ (0.42 mL, 3.83 mmol) was added slowly by a syringe on the cooled reaction mixture. The reaction mixture was slowly warmed to room temperature and stirred for 30 min, and then the reaction mixture was stirred at reflux temperature for 24h. The reaction mixture was quenched with aqueous K_2CO_3 (10%) solution. The filtration was extracted with CH_2Cl_2 (3×30 mL), and the organic layers were combined and washed with brine and dried over Na_2SO_4. The solvent was evaporated under reduced pressure, and the crude product was purified on a silica gel column chromatography using petroleum ether as eluent. The target molecule **2** (1.35 g, 74%) was isolated as a white solid (Scheme S1A). ^{1}H NMR (400 MHz, $CDCl_3$) δ 7.28-7.18 (m, =CH, 5H), 7.17-7.08 (m, =CH, 5H), 7.02 – 6.95 (m, =CH, 4H), 6.91 – 6.84 (m, =CH, 4H). ^{13}C NMR (100 MHz, $CDCl_3$) δ 142.9, 142.8, 142.4, 142.3, 140.3, 132.91, 132.90, 131.23, 131.22, 131.1, 130.9, 128.0, 127.8, 127.0, 126.9, 120.8, 120.7 (Figure S1).

*4,4'-(1,2-Diphenyl-1,2-ethenylene)dibenzaldehyde (**3**):* Compound **3** was synthesized according to the literature methods.[1-3] To a solution of **2** (1.00 g, 2.05 mmol) in anhydrous THF (50 mL) was added n-BuLi (1.25 M in hexane, 2.05 mL, 5.1 mmol) at -78 ^{o}C under nitrogen gase. After the mixture was stirred for 2h at same temperature *N*-formylpiperidine (690.0 mg, 6.1 mmol) was injected in one portion. The reaction mixture was warmed to room temperature gradually and stirred overnight at room temperature. The reaction was quenched by adding 2M aqueous hydrochloric acid (100 mL) solution. The organic layer was separated, and the aqueous layer was extracted with

Et_2O (3 × 30 mL). The organic layers were combined and dried over Na_2SO_4. After the solvent was removed under reduced pressure, the residue was purified by silica gel column chromatography using petroleum ether/ethyl acetate (9.9:0.1) as eluent to give the desired product **3** (510 mg, 64%) as a yellow solid (Scheme S1B). ^{1}H NMR (400 MHz, $CDCl_3$) δ 9.91 (s, CHO, 2H), 7.64 (dd, *J* = 8.3 Hz, =CH, 4H,), 7.20 (d, *J* = 8.3 Hz, =CH, 4H), 7.16-7.12 (m, =CH, 6H), 7.03-6.99 (m, =CH, 4H). ^{13}C NMR (100 MHz, $CDCl_3$) δ 192.0, 191.9, 149.9, 149.8, 142.5, 142.4, 140.0, 141.9, 134.9, 134.8, 132.12, 132.10, 131.43, 131.40, 129.5, 129.4, 128.4, 128.2, 127.7, 127.5 (Figure S2).

*The synthesis of **BPh-TPE:*** To a solution of 4,4'-(1,2-diphenylethene-1,2-diyl)dibenzaldehyde (**3**, 100.0 mg, 0.26 mmol) in ethanol (10 mL) was added gradually to the solution of 2-hydroxybenzohydrazide (**4**, 78.0 mg, 0.52 mmol) using dropwise. The reaction was stirred overnight at reflux with CH_3COOH (one drop) and monitored by TLC. After the completion of the reaction, the yellow product formed was recrystallized from ethanol, filtered, and dried in vacuum. After recrystallization, **BPh-Rh** (144 mg, 85%) was obtained as mixtures of E or Z isomers (Scheme S1C). **BPh-TPE**: ^{1}H NMR (400 MHz, DMSO-d6) δ 11.86 (bs, OH, NH, 4H), 8.36 (s, N=CH, 2H), 7.88 (m, =CH, 2H), 7.43-7.52 (m, =CH, 8H), 6.96-7.16 (m, =CH, 16H); ^{13}C NMR (100 MHz, $CDCl_3$) δ 165.19, 159.61, 148.73, 145.54, 143.09, 141.32, 134.30, 132.88, 131.66, 131.24, 129.03, 128.54, 128.45, 127.22, 119.35, 117.78, 116.35 (Figure S3).

*The synthesis of **BRh-TPE:*** To a solution of 4,4'-(1,2-diphenylethene-1,2-diyl)dibenzaldehyde (**3**, 100.0 mg, 0.26 mmol) in ethanol (10 mL) was added gradually to the solution of 3-amino-2-thioxothiazolidin-4-one (**5**, 76.3 mg, 0.52 mmol) using dropwise. The reaction was stirred overnight at reflux with CH_3COOH (one drop) and monitored by TLC. After the completion of the reaction, the red product formed was recrystallized from ethanol, filtered, and dried in vacuum. After recrystallization, **BRh-TPE** (134 mg, 80%) was obtained as mixtures of E or Z isomers (Scheme S1D) [2]. ^{1}H-NMR (400 MHz, DMSO-d6): δ 8.61 (s, N=CH, 2H), 7.75-7.63 (m, =CH, 8H), 7.21-6.96 (m, =CH, 10H), 4.34 (s, CH_2, 4H); ^{13}C-NMR (100 MHz, DMSO-d6): δ 197.5, 171.0, 170.3, 148.5, 142.8, 141.8, 132.2, 131.4, 13.8, 130.7, 129.2, 128.8, 127.8, 35.4 (Figure S4).

*Synthesis of **Ph-TPE** and **Np-TPE**:* Mono-substituted TPEs, **Ph-TPE** and **Rh-TPE**, were synthesized following a previously reported two-step procedure[18,21] (Scheme S1E and 1F). Starting from 4-(1,2,2-triphenylvinyl)benzaldehyde (**6**, TPE-CHO, 500 mg, 1.39 mmol), a condensation reaction with either 2-hydroxybenzohydrazide (**4**, 211 mg, 1.39 mmol) or 3-hydroxy-2-naphthohydrazide (**7**, 281 mg, 1.39 mmol) was carried out in ethanol (15 mL) under reflux conditions overnight. The resulting crude products were isolated by filtration and purified by recrystallization from ethanol, yielding **Ph-TPE** (85%) and **Rh-TPE** (72%) as yellow solids. Detailed experimental procedures and spectroscopic data are provided in the supplementary information. **Ph-TPE:** ^{1}H NMR (400 MHz, DMSO-d6) δ 11.87 (bs, OH, 1H), 11.83 (bs, NH, 1H), 8.35 (s, N=CH,

1H), 7.88 (d, *J* = 8.1 Hz, =CH, 1H), 7.50 (m, A part of AB system, =CH, 2H), 7.43 (t, *J* = 8.1 Hz, =CH, 1H), 7.11-7.16 (m, =CH, 9H), 7.05 (m, B part of AB system, =CH, 2H), 6.93-7.00 (m, =CH, 8H); ^{13}C NMR (100 MHz, $CDCl_3$) δ 165.17, 159.51, 148.76, 145.76, 143.44 (2C), 143.26, 141.86, 140.48, 134.33, 132.70, 131.66, 131.19, 131.17, 131.12, 129.02, 128.43 (2C), 128.33, 127.28, 127.22 (3C), 119.43, 117.43, 116.31; (Figure S5). **Rh-TPE:** ^{1}H NMR (400 MHz, DMSO-d6) δ 11.97 (bs, OH, 1H), 11.33 (bs, NH, 1H), 8.44 (s, N=CH, 1H), 8.36 (s, =CH, 1H), 7.91 (d, *J* = 8.2 Hz, =CH, 1H), 7.76 (d, *J* = 8.2 Hz, =CH, 1H), 7.52 (m, A part of AB system, =CH, 2H), 7.32-7.38 (m, =CH, 2H), 7.11-7.16 (m, =CH, 10H), 7.06 (m, B part of AB system, =CH, 2H), 6.98-7.01 (m, =CH, 6H); ^{13}C NMR (100 MHz, $CDCl_3$) δ 164.21, 154.59, 148.63, 145.76, 143.45 (2C), 143.26, 141.88, 140.51, 136.34, 132.76, 131.66, 131.19 (2C), 131.16, 131.11, 130.74, 129.15, 128.42, 128.32 (2C), 127.28, 127.22 (3C), 127.17, 126.32, 124.28, 120.69, 111.07 (Figure S6).

UV-Vis and fluorescence studies of bis-substituted-TPEs with various ions: The solution of bis-substituted-TPEs (1×10^{-2} M) and ions (1×10^{-2} M) were prepared in EtOH and H_2O, respectively. A solution of bis-substituted-TPEs (10 µM) was placed in a quartz cell and the UV-Vis and fluorescence spectrums were recorded in water. After introduction of the solution of ions (1 equiv.), the changes in absorbance intensity were recorded at room temperature each time.

UV-Vis and fluorescence titration of bis-substituted-TPEs with $CuCl_2$ / $HgCl_2$ / AgCl / GSH: The solution of probe bis-substituted-TPEs (1×10^{-2} M) and $CuCl_2$ / $HgCl_2$ / AgCl / GSH (1×10^{-2} M) were prepared in EtOH and H_2O, respectively. The concentration of probe bis-substituted-TPEs used in the experiments was 10 µM. The UV-Vis and fluorescence titration spectras were recorded by adding the corresponding CuCl2 / HgCl2 / AgCl / GSH concentration to a solution of bis-substituted TPEs in water. Each titration was repeated at least twice until consistent values were obtained.

Job's plot measurement: Probes bis-substituted-TPEs were dissolved in water to make the concentration of 1×10^{-2} M. 5.00, 4.50, 4.00, 3.50, 3.00, 2.50, 2.00, 1.50, 1.00, 0.50 and 0.0 mL of the ligand solution were taken and transferred to vials. $CuCl_2$ / $HgCl_2$ / AgCl was dissolved in H_2O to make the concentration of 1×10^{-2} M. 0.0, 0.50, 1.00, 1.50, 2.00, 2.50, 3.00, 3.50, 4.00, 4.50, and 5 mL of the $CuCl_2$ / $HgCl_2$ / AgCl solution were added to each ligand solution. Each vial had a total volume of 5 mL. After shaking the vials for a few seconds, absorbance spectras were taken at room temperature.

Determination of detection limit: The fluorescence measurements were taken for each solution containing $CuCl_2$ / $HgCl_2$ / AgCl / GSH. The detection limit for $CuCl_2$ / $HgCl_2$ / AgCl / GSH was calculated based on the fluorescence titration. For this purpose, **3s/k** equation was used. Where *s* is the standard deviation of blank, *k* is the slope of the fit line in the fluorescence or absorbance titration experiment.

Determination of Association Constant: Association constant was calculated according to the Benesi-Hildebrand equation by fluorescent method. Association Constant (K_a) was calculated following the equation stated below:

$$\frac{1}{F-F_0} = \frac{1}{\{K_a(F_{max}-F_0)[M^{x+}]^n\}} + \frac{1}{F_{max}-F_0}$$

Here, F_o is the fluorescence of receptor in the absence of metal ion, *F* is the fluorescence recorded in the presence of added metal ion, F_{max} is fluorescence in presence of added $[M^{x+}]$ max and K_a is the association constant, where $[M^{x+}]$ is $[M^{2+ \text{ or } +}]$. n is the binding stoichiometry for receptor and metal ion. The association constant (K_a) could be determined from the slope of the straight line of the plot of $1/F_o\text{-}F$ against $1/[M]^{1/2}$.

The pH measurement: The effect of different pH environments (range of 2–12) was studied for the practical application of the bis-substituted-TPEs (10 μM, in EtOH) in the absence and presence of $CuCl_2$ / $HgCl_2$ / AgCl (30 μM, in H_2O). For this purpose, the bis-substituted-TPEs samples were prepared in ten different tubes, and $CuCl_2$ / $HgCl_2$ / AgCl ions dissolved in water were added. The pH values of samples were modulated by adding HCl or NaOH solution. pH values of the solution were monitored with a pH meter and/or pH stick.

Electronic Properties:

Through the medium of HOMO and LUMO energies, electronic properties, electron affinity (EA), ionization potential (IP), chemical hardness (η), chemical softness (ζ), electronegativity (χ), chemical potential (μ), global electrophilicity index (ω), maximum charge transfer index (ΔNmax) can be defined as follows; [4-9]

$EA = -E_{LUMO}$

$IP = -E_{HOMO}$

$\eta = {}^{1}/_{2}\,(E_{LUMO} - E_{HOMO})$

$\zeta = \frac{1}{\eta}$

$\chi = -\,{}^{1}/_{2}\,(E_{LUMO} + E_{HOMO})$

$\mu = {}^{1}/_{2}\,(E_{LUMO} + E_{HOMO})$

$\omega = \frac{\mu^2}{2\eta}$

$\Delta N_{max} = -\frac{\mu}{\eta}$

$N = \frac{4(IP - EA)}{(IP + EA)^2}$

$\sigma_{\circ} = \frac{1}{IP - EA}$

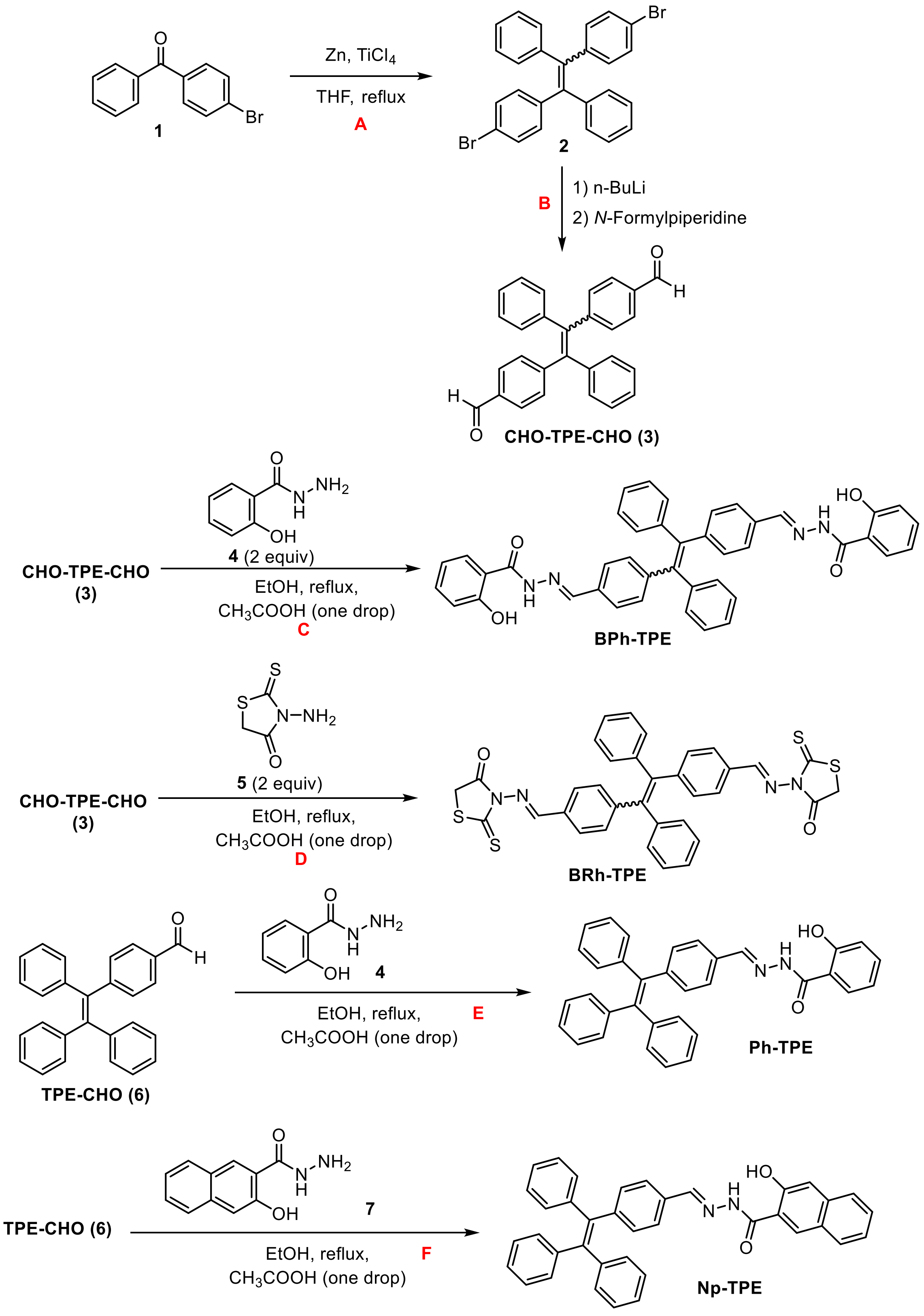

Scheme S1. Synthesis strategies of TPEs

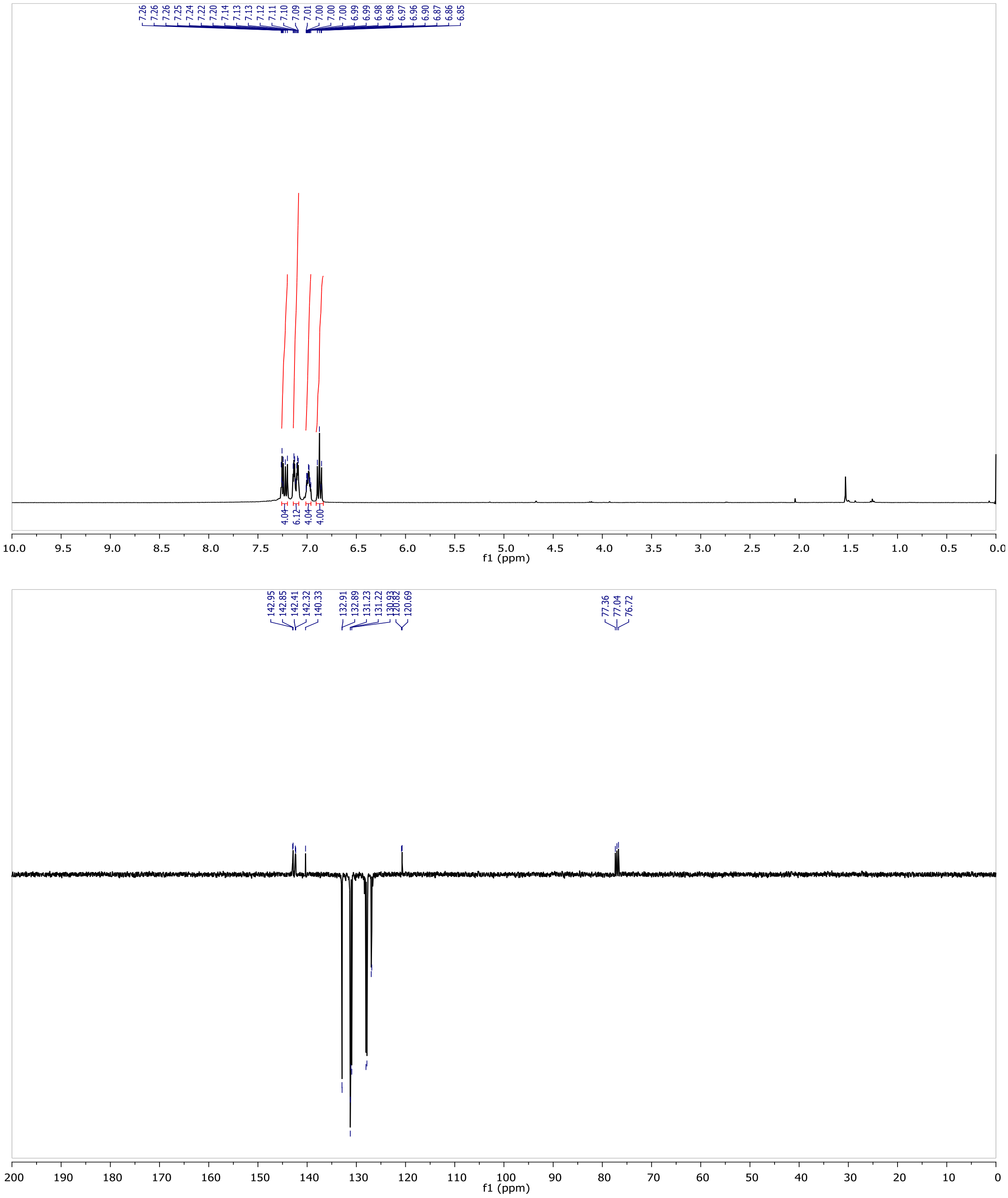

Figure S1. The NMR spectrums of 1,2-Bis(4-bromophenyl)-1,2-diphenylethene (**2**) in $CDCl_3$.

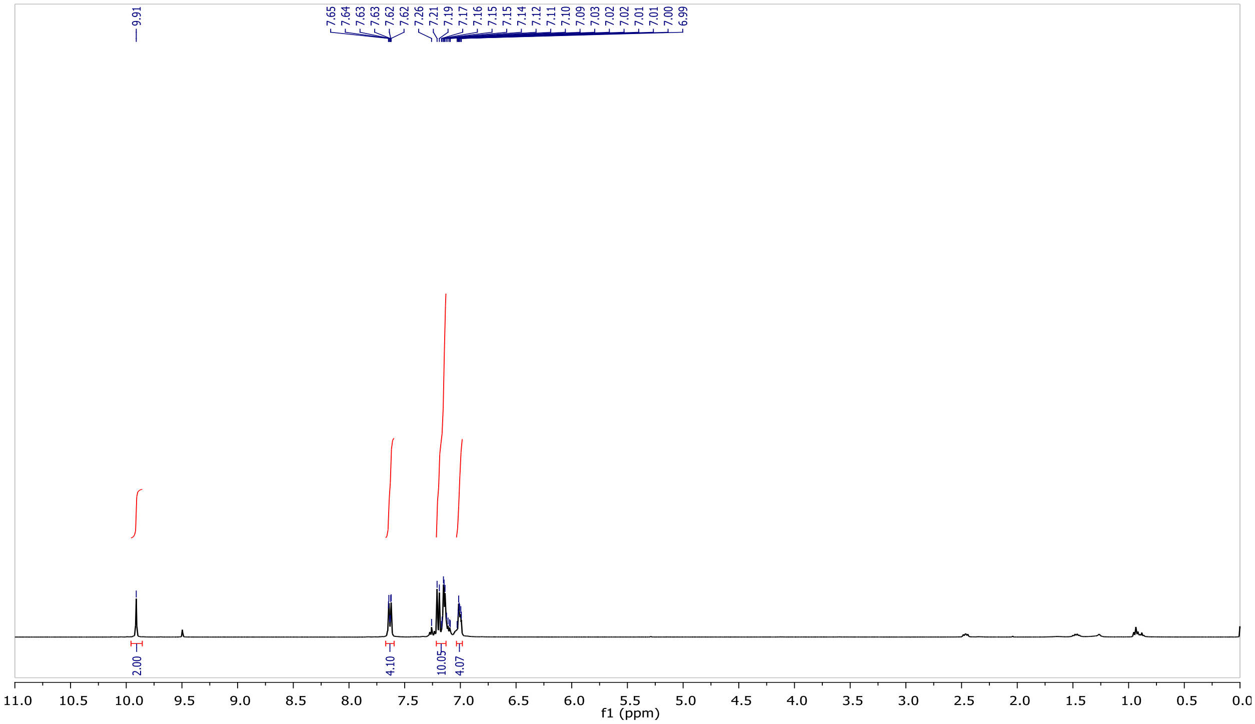

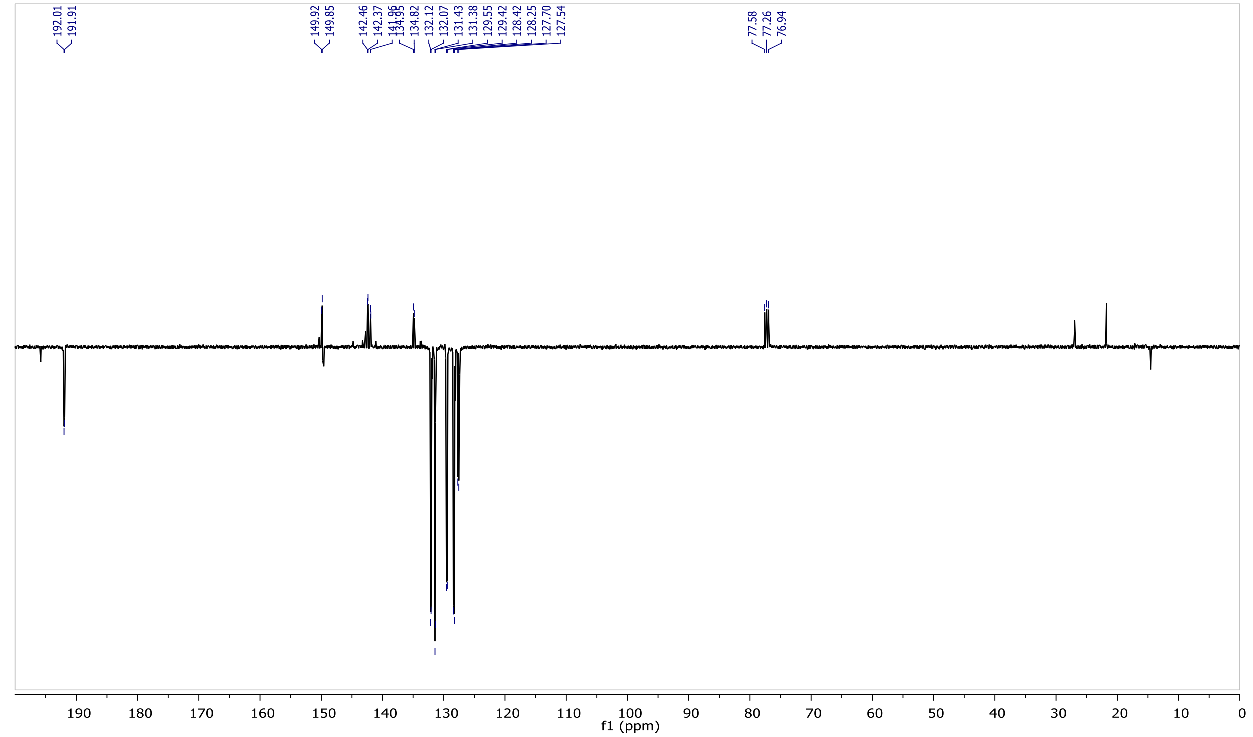

Figure S2. The NMR spectrums of 4,4′-(1,2-Diphenyl-1,2-ethenylene)dibenzaldehyde (**3**) in $CDCl_3$

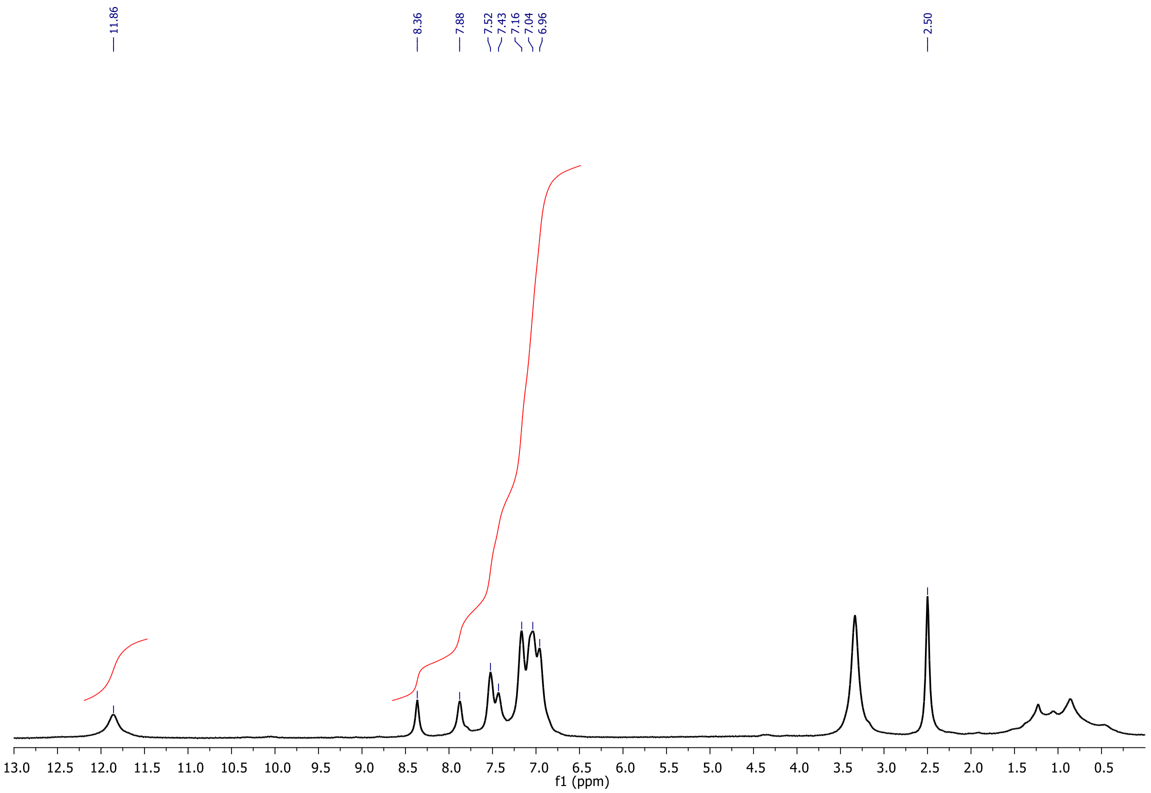

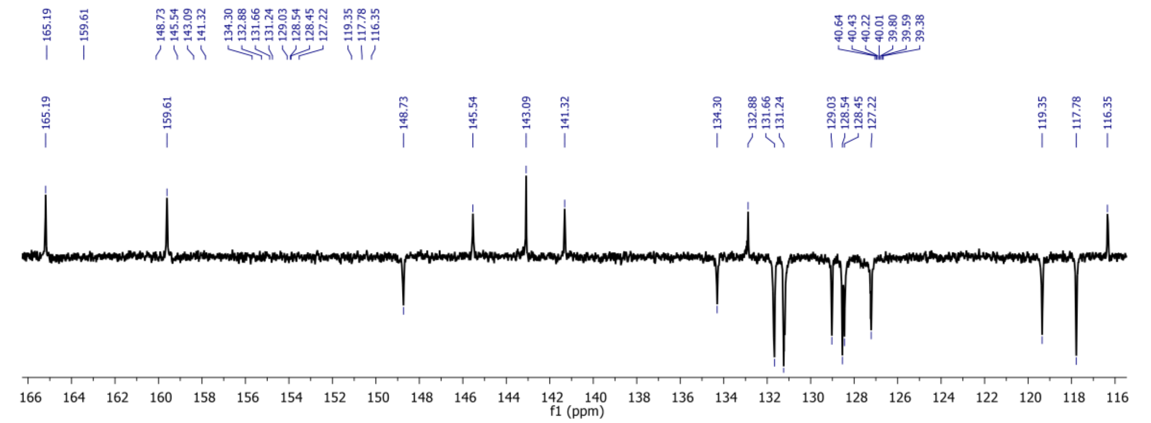

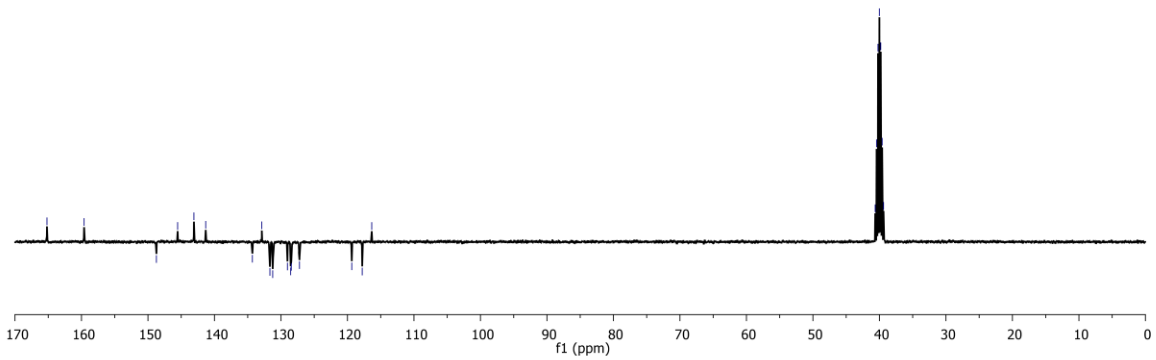

Figure S3. The NMR spectrums of BPh- TPE in DMSO-d6

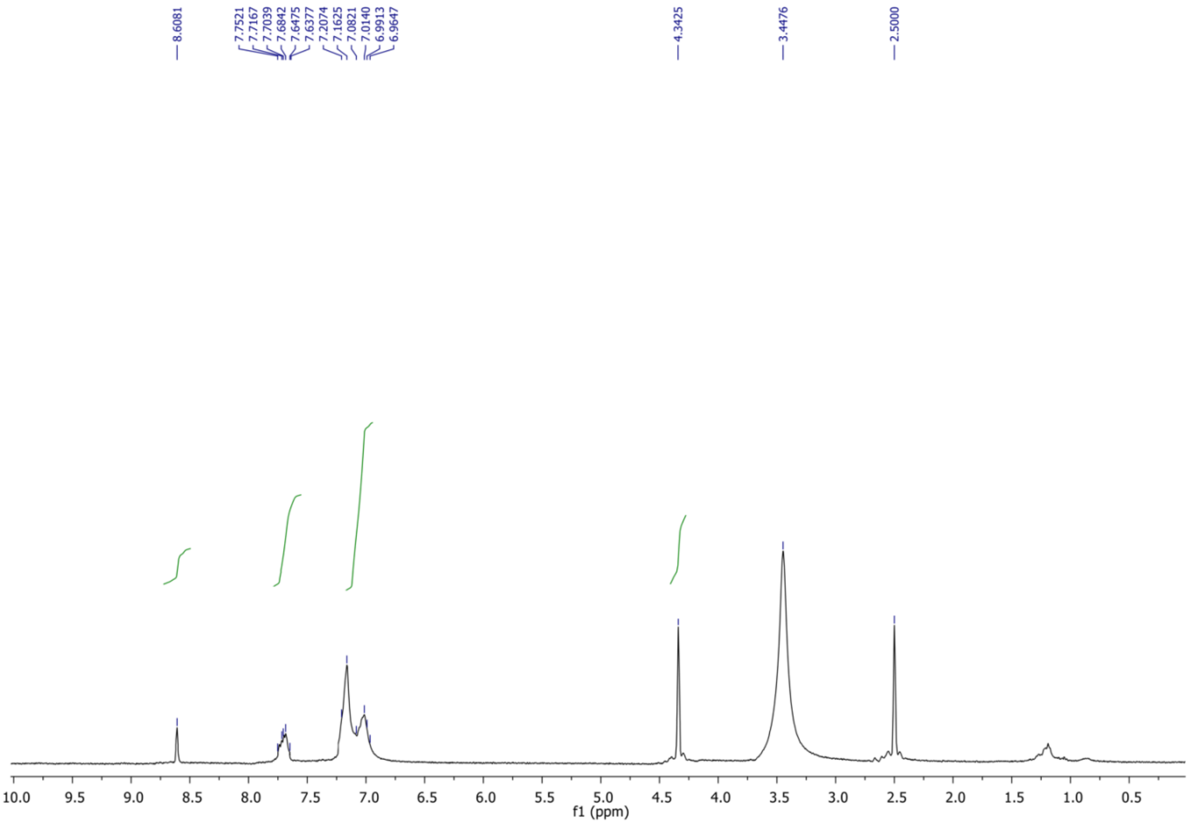

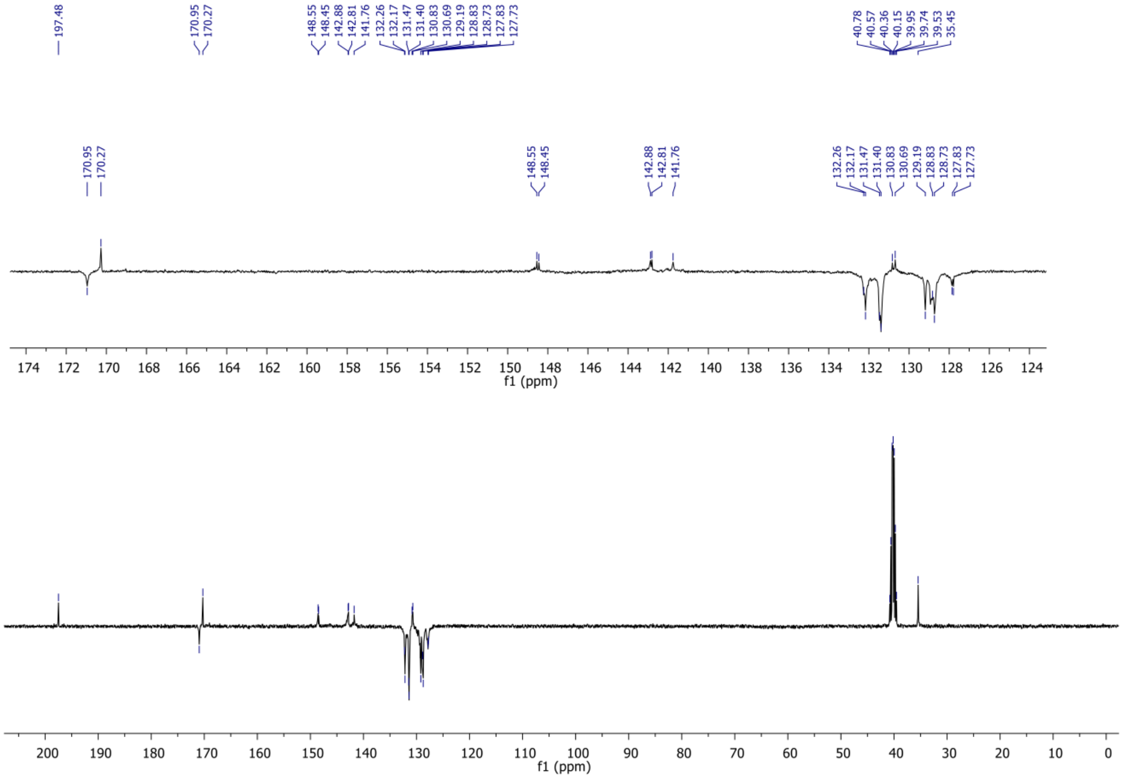

Figure S4. The NMR spectrums of BRh- TPE in DMSO-d6

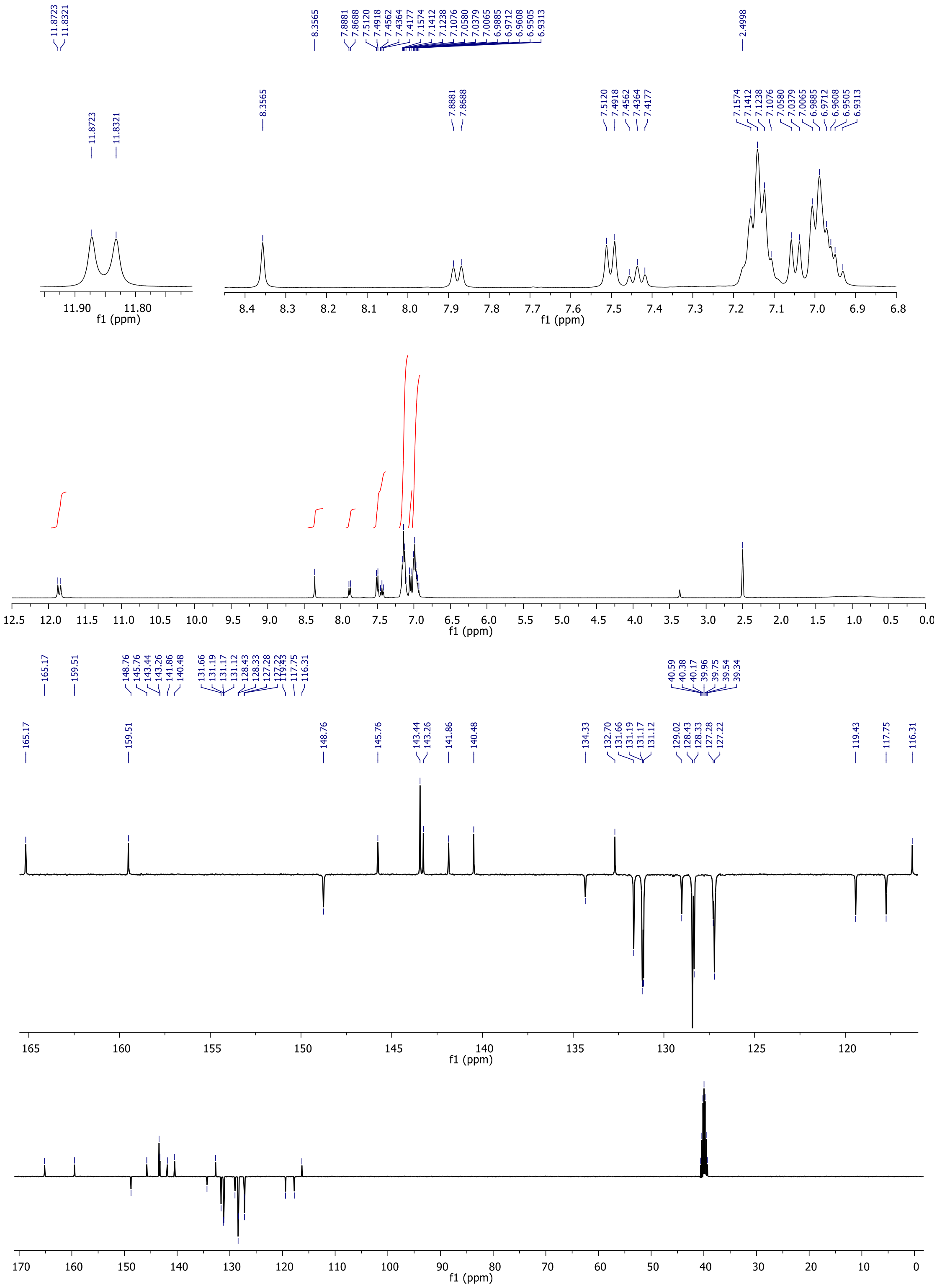

Figure S5. NMR spectrums of Ph-TPE in DMSO-d6.

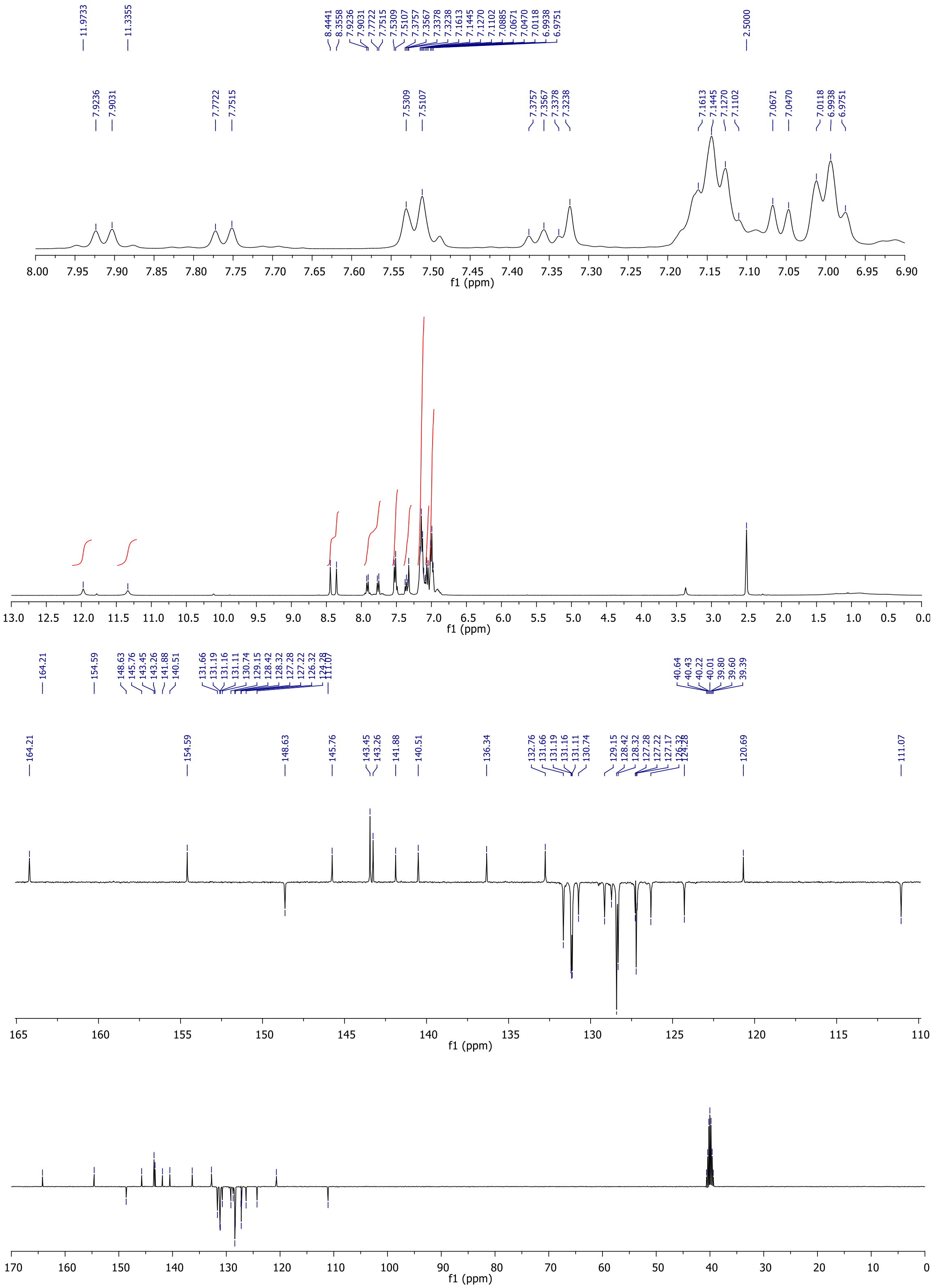

Figure S6. NMR spectrums of Np-TPE in DMSO-d6.

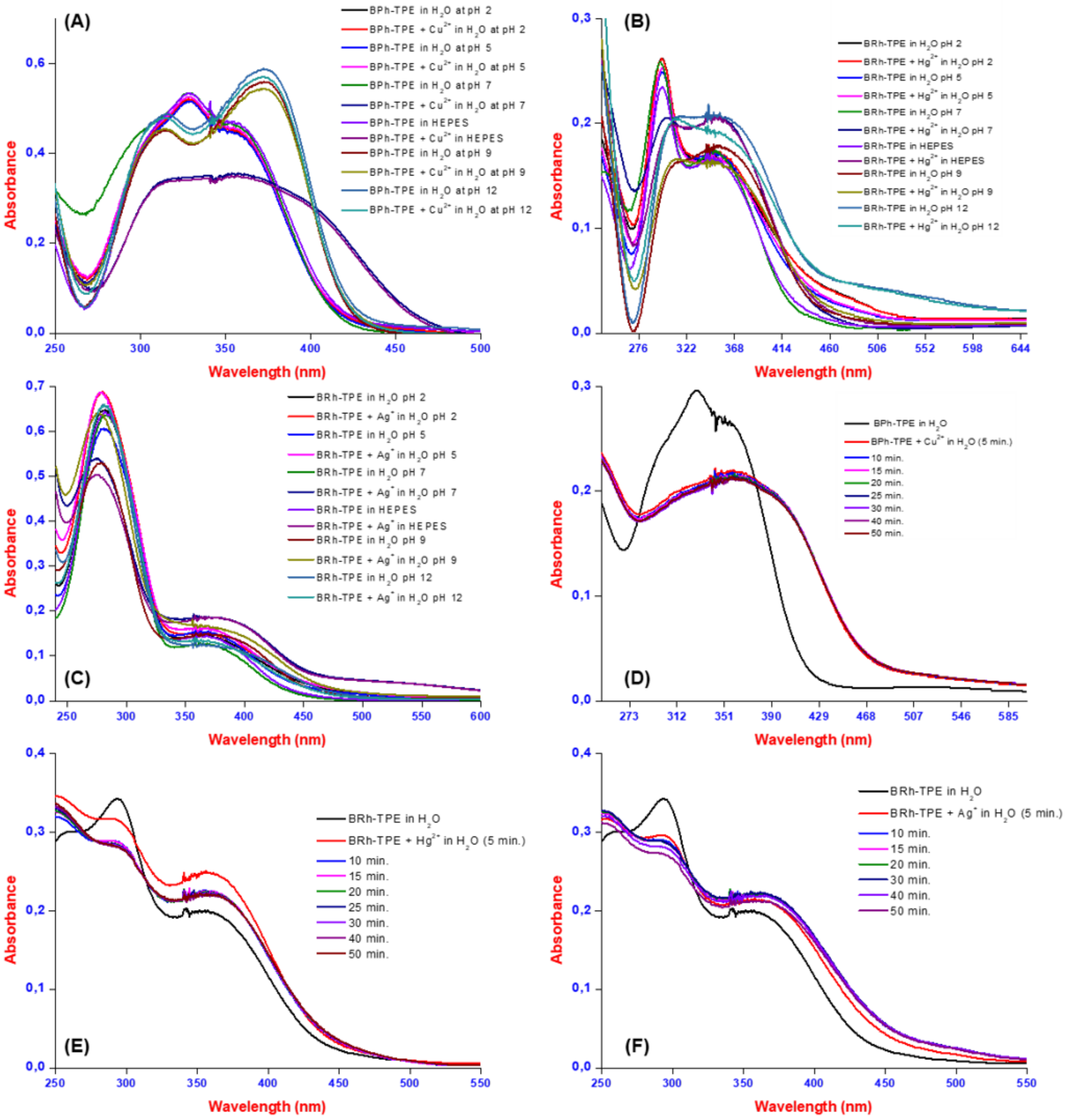

Figure S7. (**A**-**C**) The absorbance spectras of bis-substituted-TPEs with [$CuCl_2$] / [$HgCl_2$] / [AgCl] at different pH (2–12) in water, the pH was modulated by adding 75% HCl or NaOH solution. (**D**-**F**) Absorbance enhancing profile of addition Cu^{2+} / Hg^{2+} / Ag^{+} to bis-substituted-TPEs in water from 10 to 60 minutes.

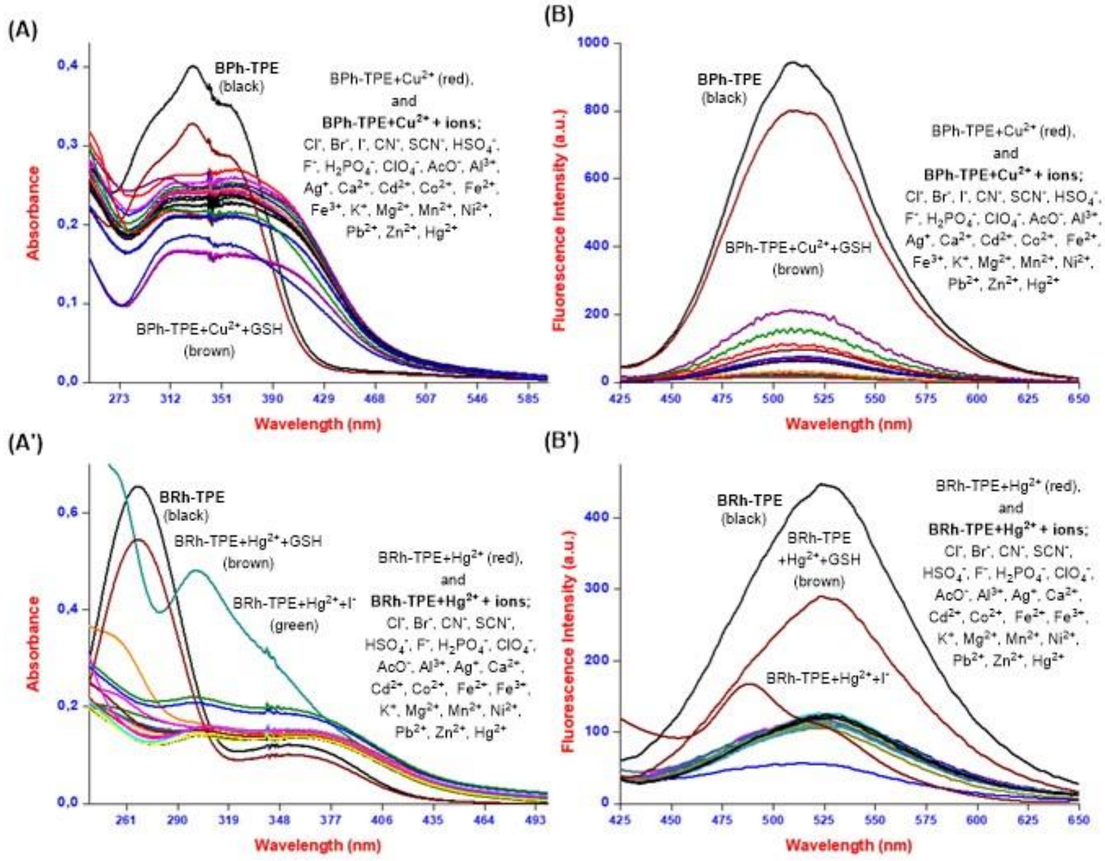

Figure S8. Absorbance and fluorescence spectra of BPh-TPE-Cu^{2+} (**A** and **B**) and BRh-TPE-Hg^{2+} (**C** and **D**) upon adding different ions in water up to 1 equivalent.

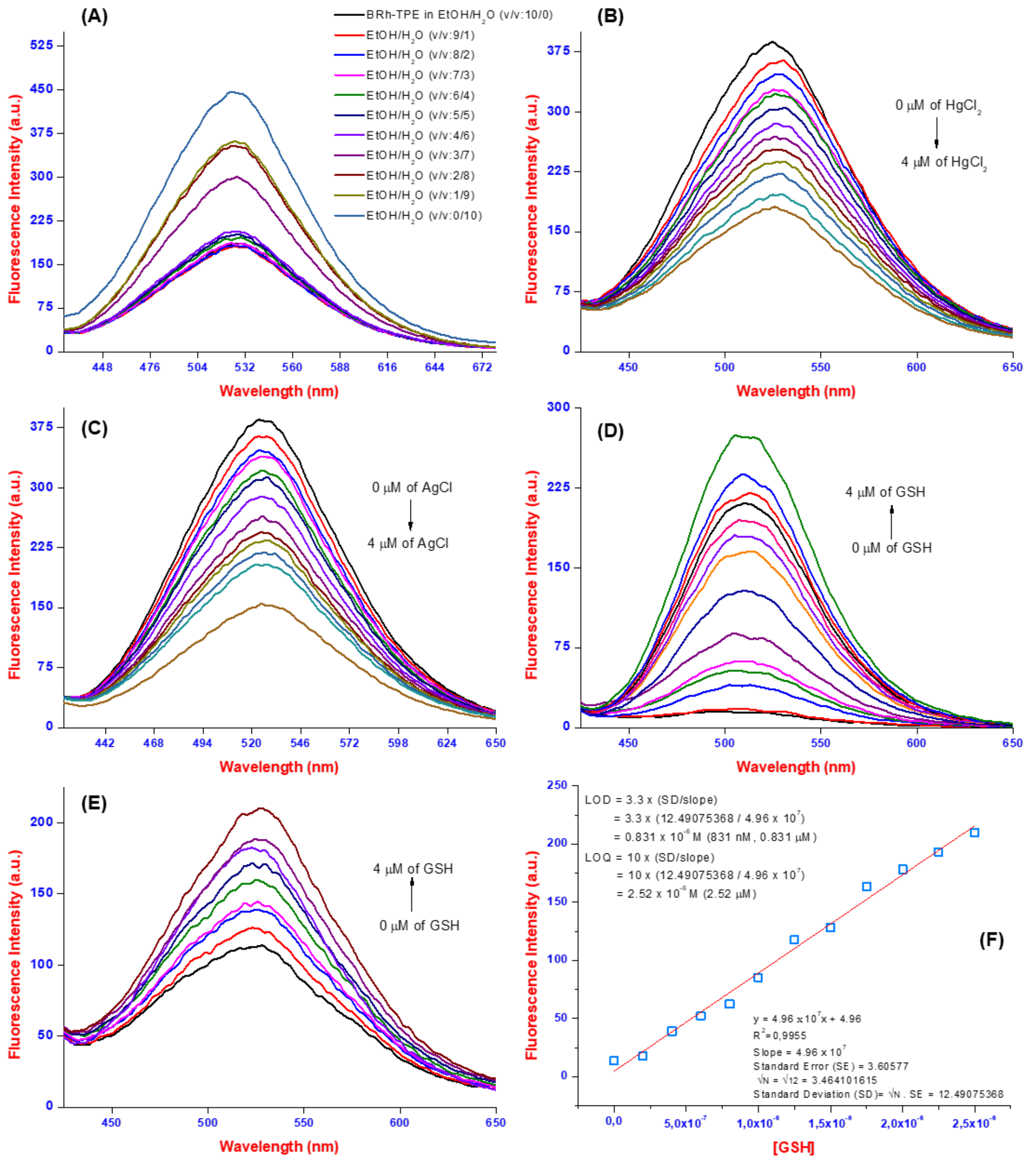

Figure S9. (**A**) The AIE fluorescent response of BRh-TPE in different water ratio mixtures. The fluorescence titration spectra of (**B**) BRh-TPE with Hg^{2+},(**C**) BRh-TPE with Ag^{+}, (**D**) BPh-TPE-Cu^{2+} with GSH and (**E**) BRh-TPE-Hg^{2+} with GSH in water. (**F**) The change fluorescence intensity of BRh-TPE-Hg^{2+} with the increasing concentration of GSH in water.

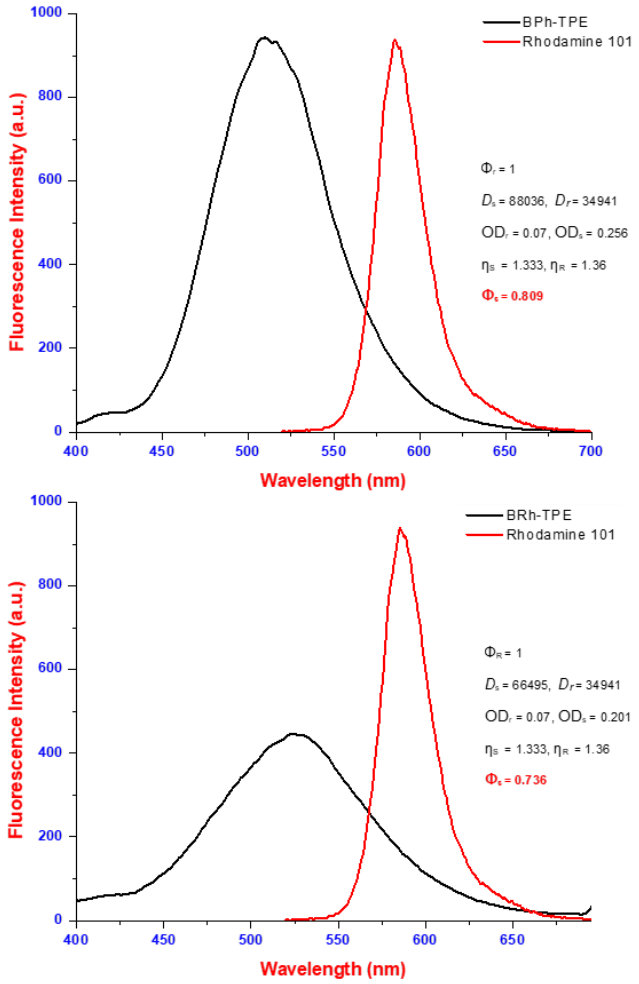

Figure S10. Fluorescence spectras of bis-substituted-TPEs and rhodamine 101 for the fluorescence quantum yield

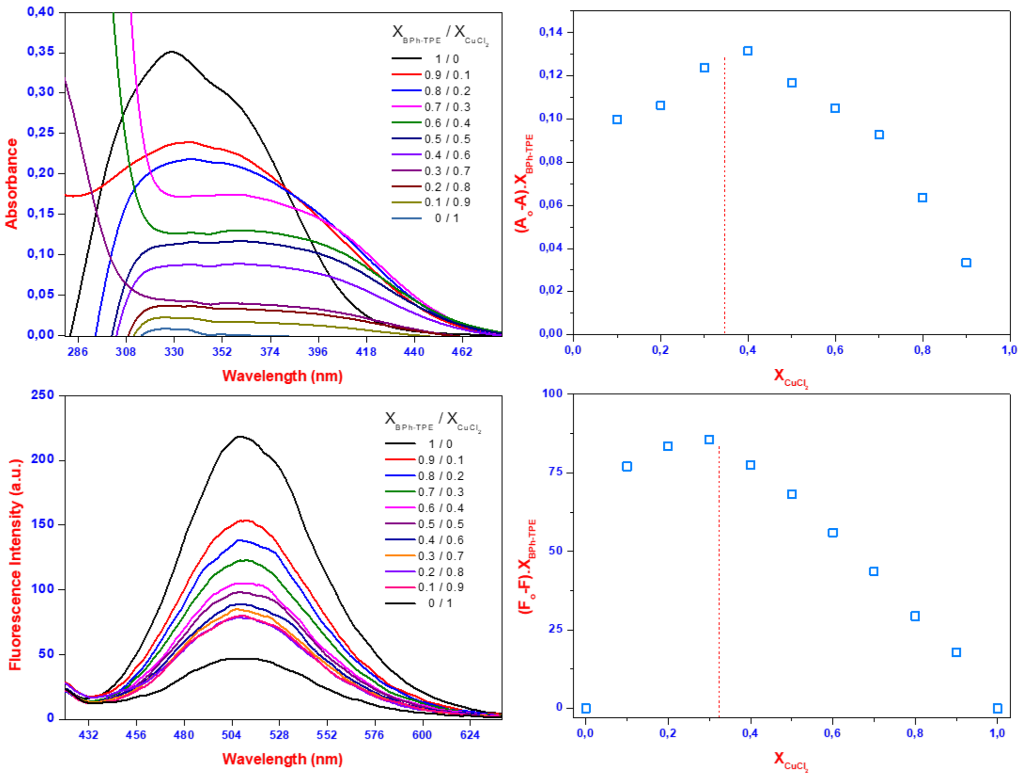

Figure S11. The Job plot absorbance and fluorescence spectras of BPh-TPE with $CuCl_2$ in water

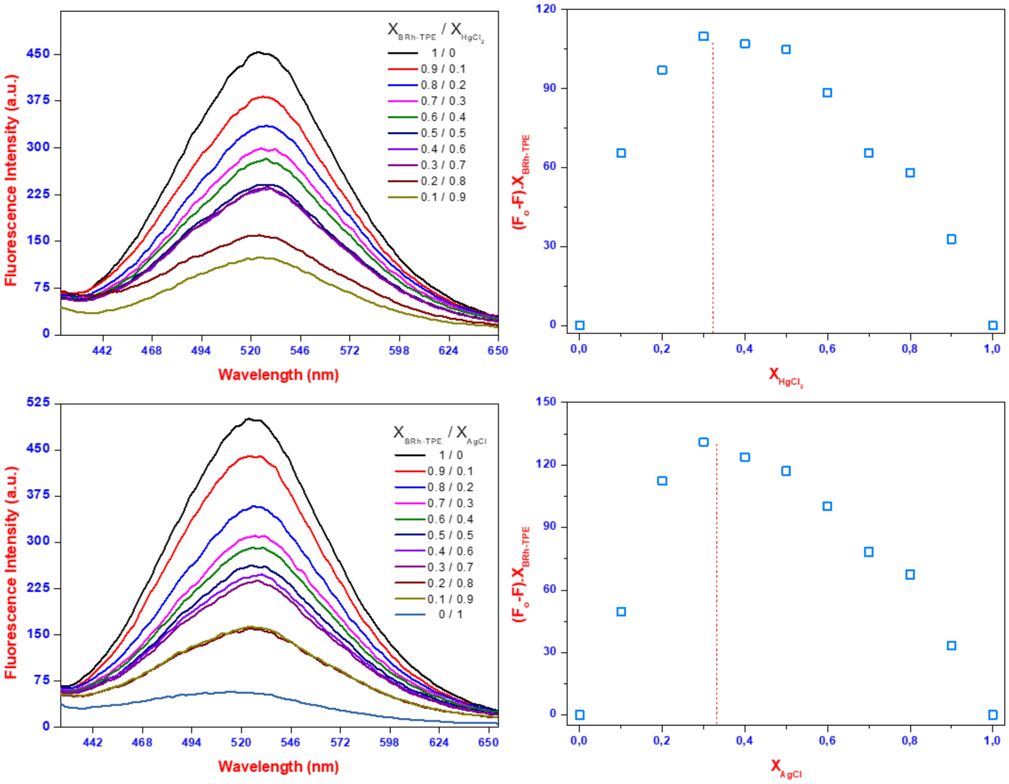

Figure S12. The Job plot fluorescence spectras of BRh-TPE with $HgCl_2$ / AgCl in water

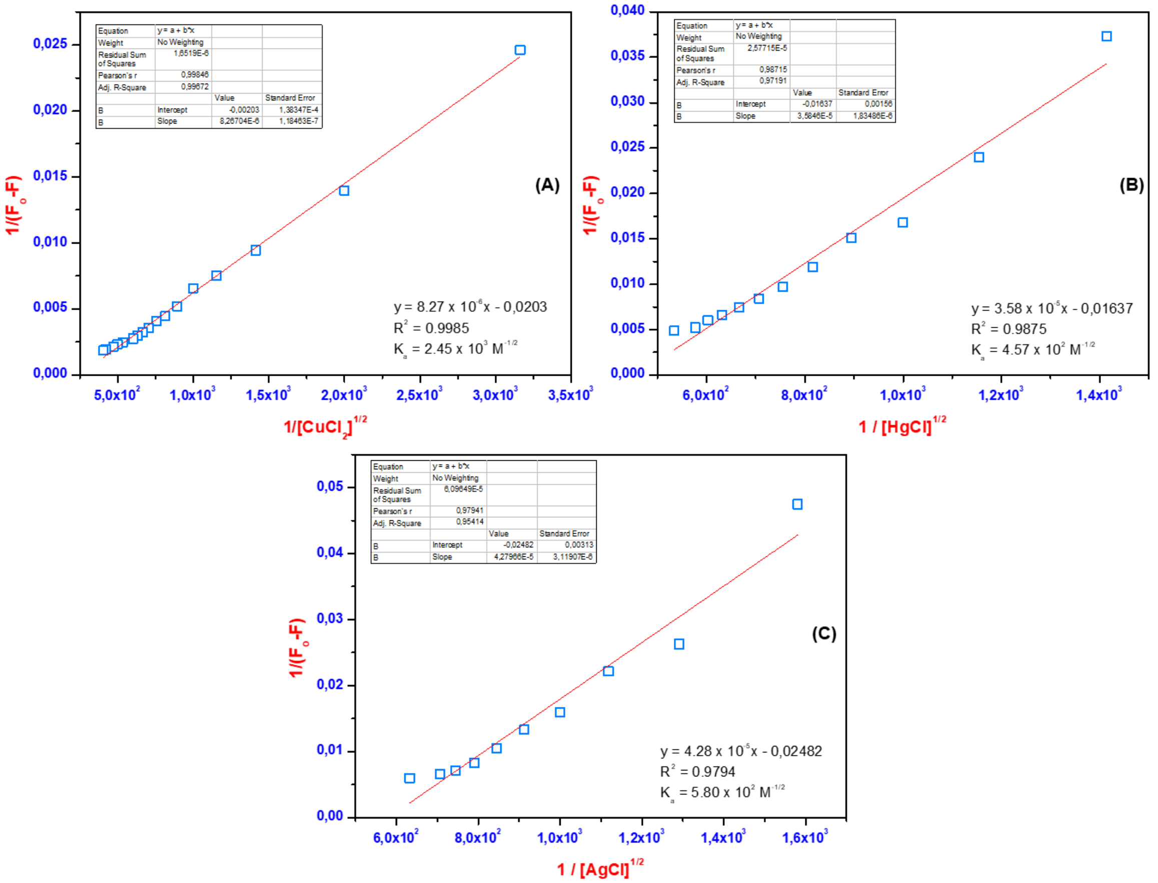

Figure S13. Benesi–Hildebrand plot based on a 1:2 association stoichiometry between TPEs with Cu^{2+} (**A**), Hg^{2+} (**B**), and Ag^{+} (**C**) ions.

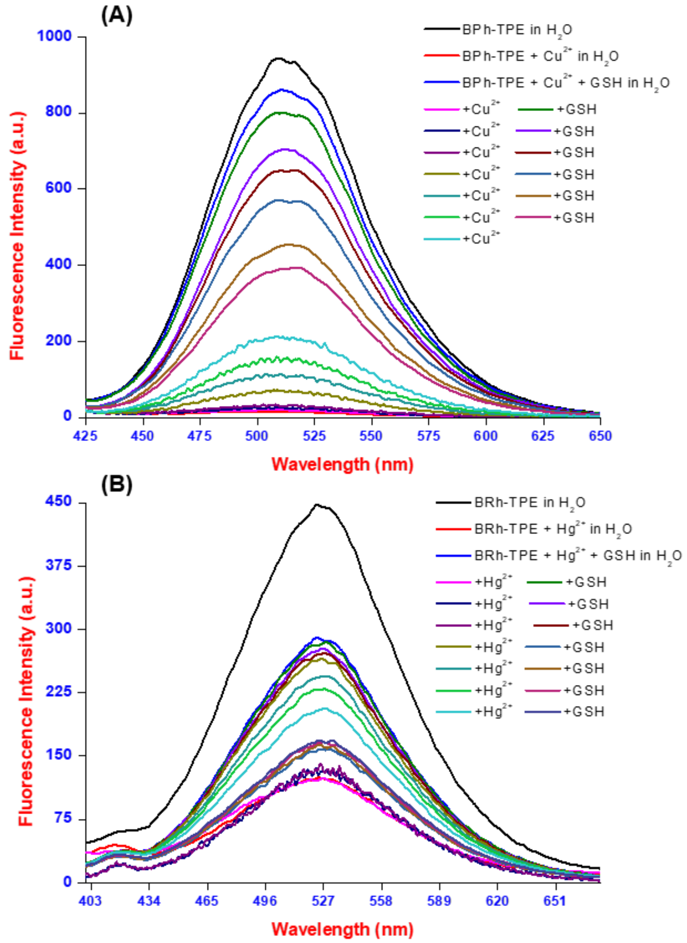

Figure S14. Reversible switching of the fluorescence spectrum of BPh-TPE (**A**) and BRh-TPE (**B**) with alternate addition of Cu^{2+} / Hg^{2+} and GSH in water.

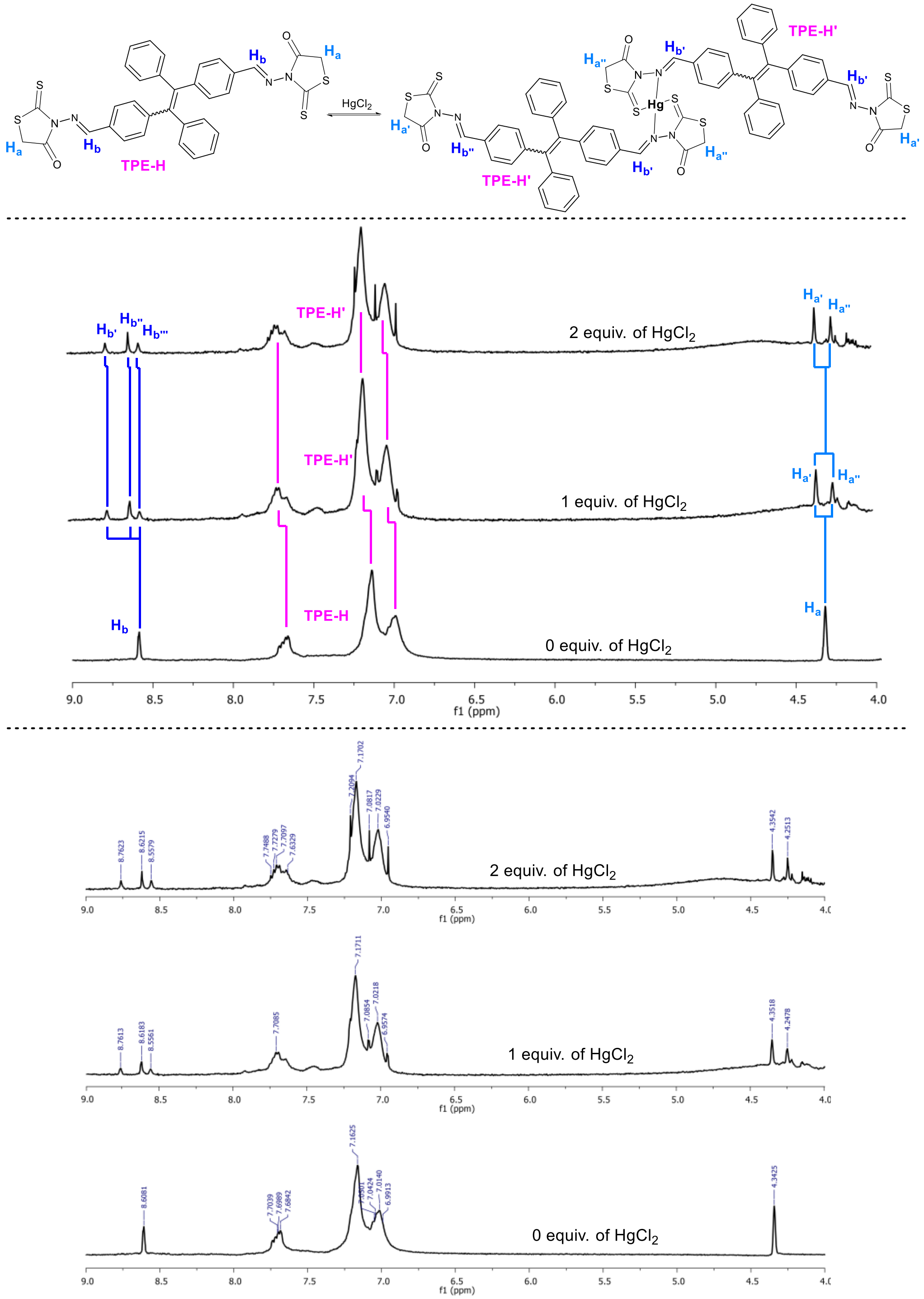

Figure S15. Change in partial ^{1}H-NMR (400 MHz) spectra of BRh-TPE with $HgCl_2$ in DMSO-d6.

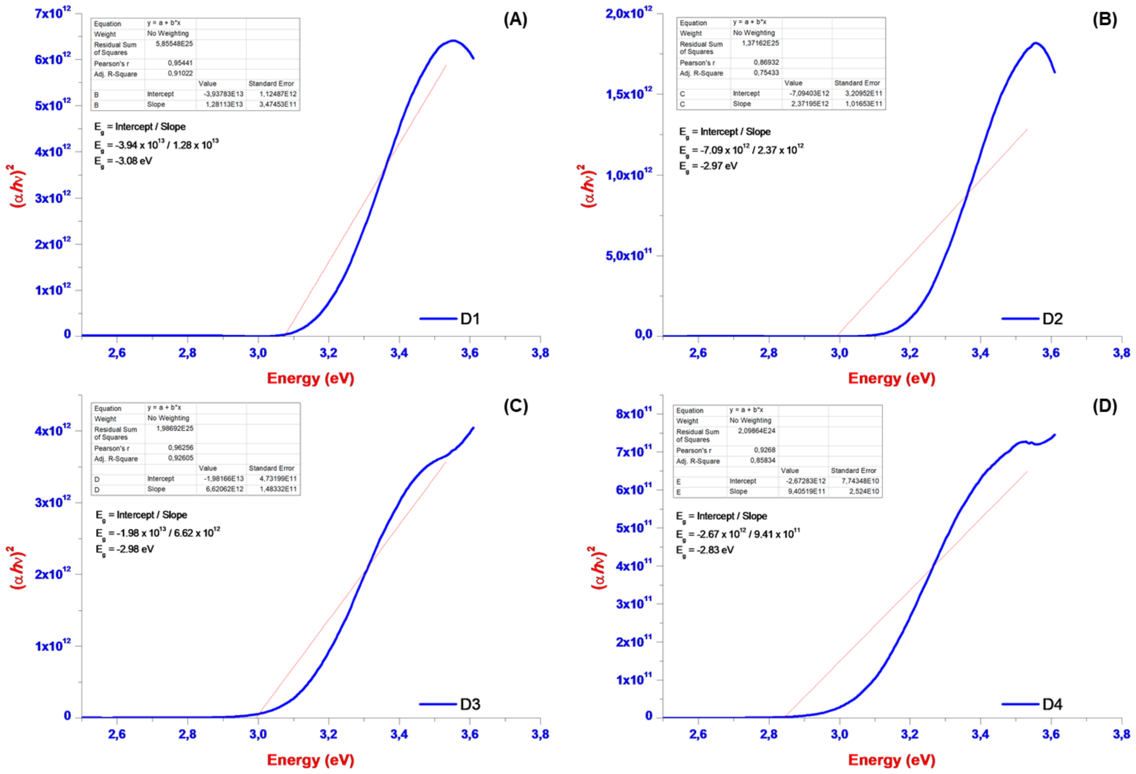

Figure S16. The band-gap energies details of D1 (**A**), D2 (**B**), D3 (**C**) and D4 (**D**) devices.

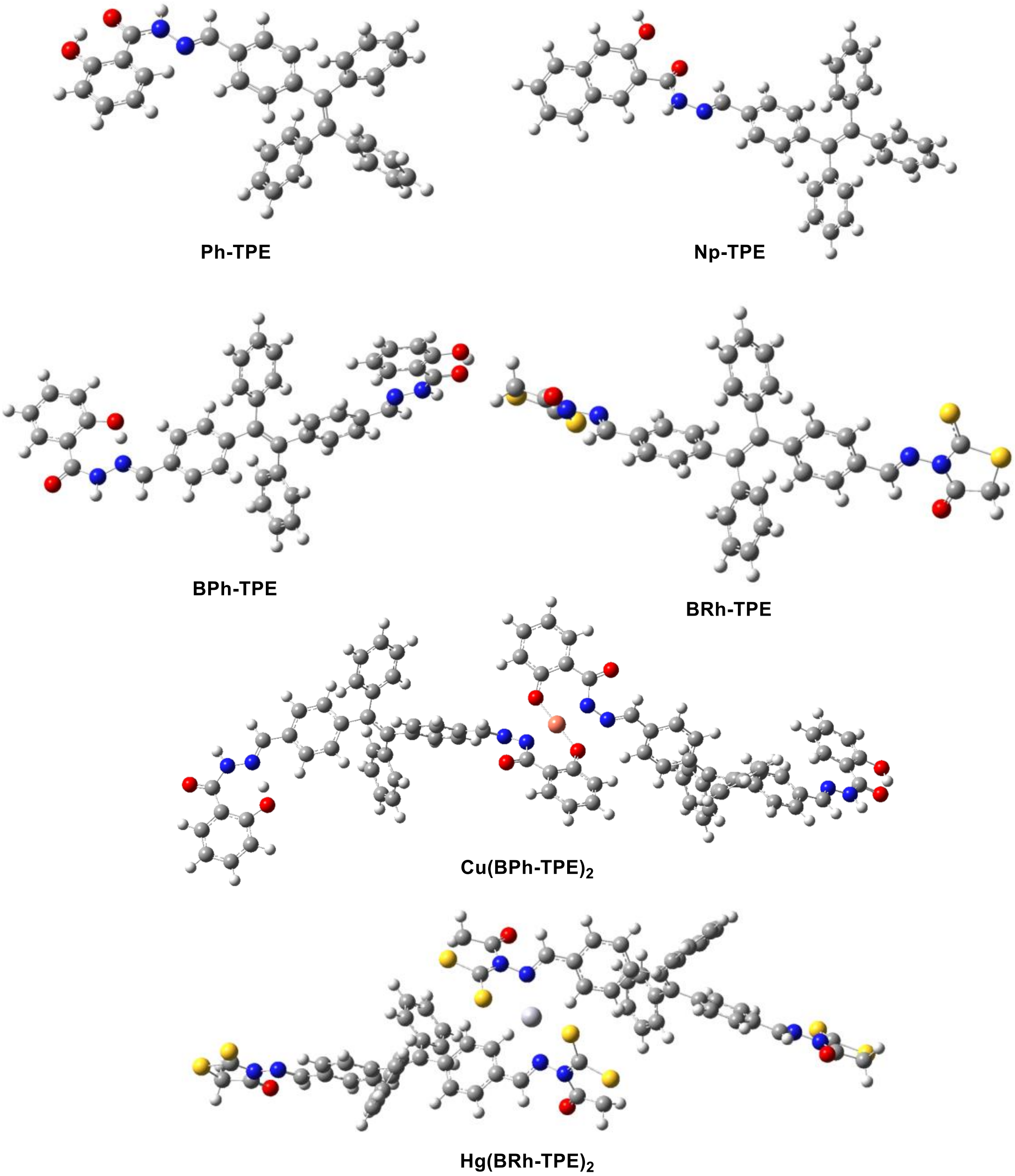

Figure S17. The optimized molecular geometries of TPEs

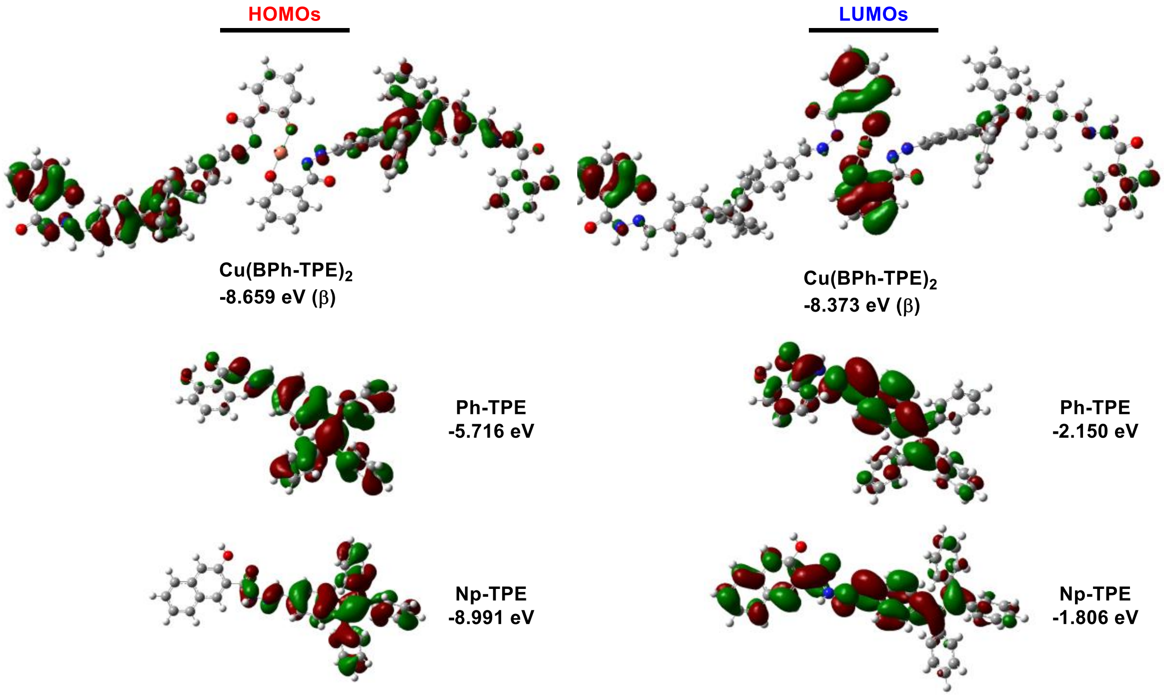

Figure S18. The HOMO/LUMO orbital distributions of TPEs

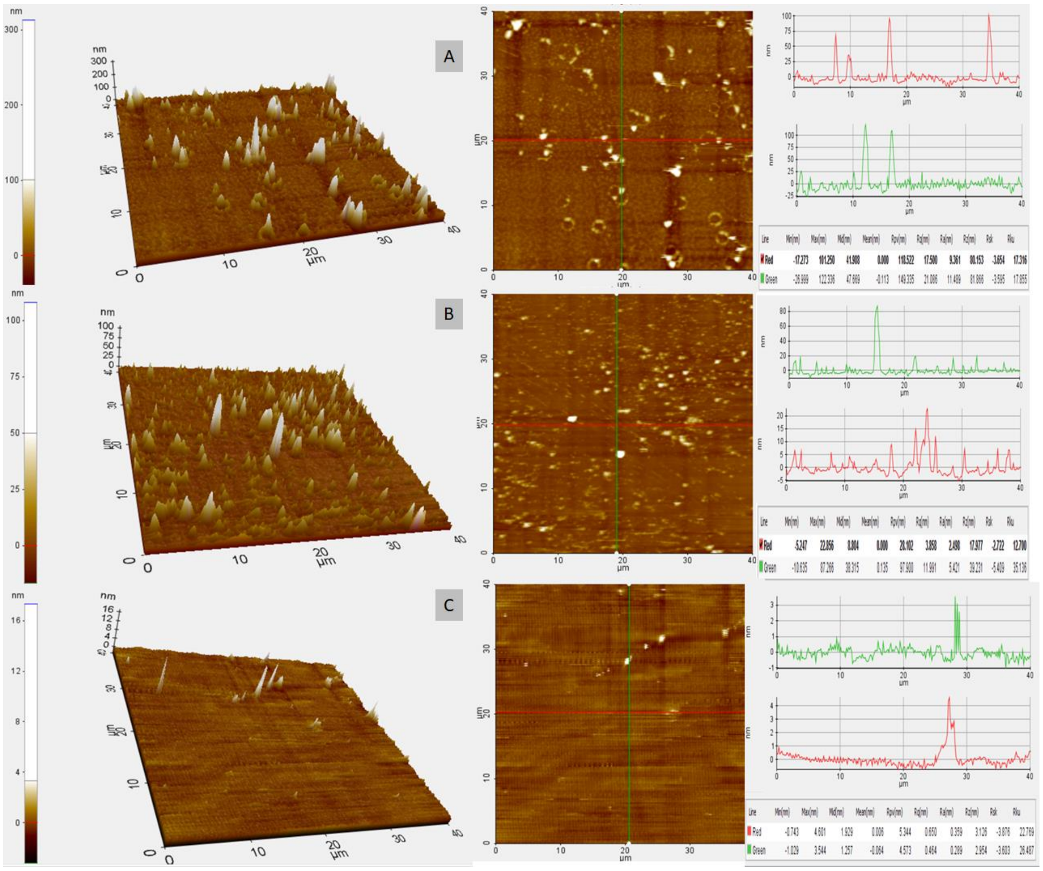

Figure S19. 2D and 3D AFM measurements of D1, D2 and D3

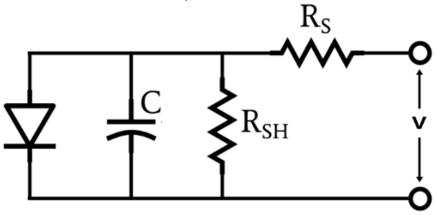

Figure S20. An equivalent circuit model diode

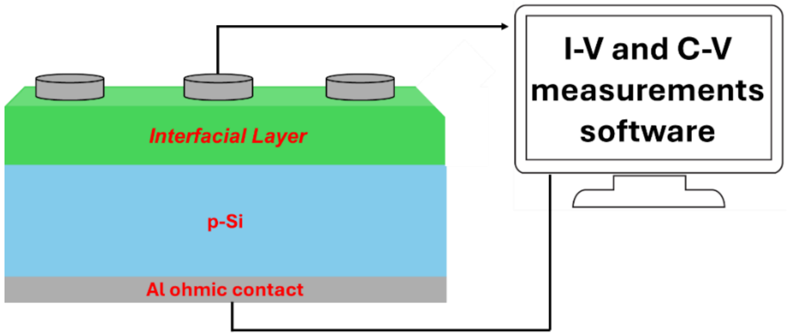

Figure S21. Schematic diagram of devices

Referance

1. A. Karabulut, F. Lafzi, S. Bayindir, Ö. Sevgili, I. Orak, Journal of Molecular Structure 2021, 1231, 129699.

2. S. Bayindir, A. S. Hussein, F. Lafzi, M. Toprak, Journal of Molecular Liquids 2023, 382, 121939.

3. Ö. Sevgili, F. Lafzi, A. Karabulut, I. Orak, S. Bayındır, Composites Part B: Engineering 2019, 172, 226-233.

4. R. G. Parr, R. A. Donnelly, M. Levy, W. E. Palke, The Journal of chemical physics 1978, 68, 3801-3807.

5. P. Politzer, H. Weinstein, The Journal of chemical physics 1979, 71, 4218-4220.

6. R. G. Parr, W. Yang, Journal of the American Chemical Society 1984, 106, 4049-4050.

7. P. K. Chattaraj, B. Maiti, U. Sarkar, The Journal of Physical Chemistry A 2003, 107, 4973-4975.

8. R. G. Parr, L. v. Szentpály, S. Liu, Journal of the American Chemical Society 1999, 121, 1922-1924.

9. J. Padmanabhan, R. Parthasarathi, V. Subramanian, P. Chattaraj, The Journal of Physical Chemistry A 2007, 111, 1358-1361.
